# Precision medicine in human heart modeling: Perspectives, challenges, and opportunities

**DOI:** 10.1007/s10237-021-01421-z

**Published:** 2021-02-12

**Authors:** M. Peirlinck, F. Sahli Costabal, J. Yao, J. M. Guccione, S. Tripathy, Y. Wang, D. Ozturk, P. Segars, T. M. Morrison, S. Levine, E. Kuhl

**Affiliations:** 1 Department of Mechanical Engineering, Stanford University, Stanford, California USA; 2 Department of Mechanical Engineering, Pontificia Universidad Catholica de Chile, Santiago, Chile; 3 Dassault Systèmes Simulia Corporation, Johnston, Rhode Island USA; 4 University of California, San Francisco, California USA; 5 Edwards Lifesciences, Irvine, California USA; 6 Thornton Tomasetti Inc., Santa Clara, California USA; 7 Capvidia, Leuven, Belgium; 8 Carl E. Ravin Advanced Imaging Laboratories, Department of Radiology, Duke University, Durham, North Carolina USA; 9 Center for Devices and Radiological Health, U.S. Food and Drug Administration, Silver Spring, Maryland USA; 10 Departments of Mechanical Engineering and Bioengineering, Stanford University, Stanford, California USA

**Keywords:** Precision medicine, Electrophysiology, Cardiac mechanics, Fluid dynamics, Finite element simulation, Machine learning, Digital twin

## Abstract

Precision medicine is a new frontier in healthcare that uses scientific methods to customize medical treatment to the individual genes, anatomy, physiology, and lifestyle of each person. In cardiovascular health, precision medicine has emerged as a promising paradigm to enable cost-effective solutions that improve quality of life and reduce mortality rates. However, the exact role in precision medicine for human heart modeling has not yet been fully explored. Here, we discuss the challenges and opportunities for personalized human heart simulations, from diagnosis to device design, treatment planning, and prognosis. With a view toward personalization, we map out the history of anatomic, physical, and constitutive human heart models throughout the past three decades. We illustrate recent human heart modeling in electrophysiology, cardiac mechanics, and fluid dynamics and highlight clinically relevant applications of these models for drug development, pacing lead failure, heart failure, ventricular assist devices, edge-to-edge repair, and annuloplasty. With a view toward translational medicine, we provide a clinical perspective on virtual imaging trials and a regulatory perspective on medical device innovation. We show that precision medicine in human heart modeling does not necessarily require a fully personalized, high-resolution whole heart model with an entire personalized medical history. Instead, we advocate for creating personalized models out of population-based libraries with geometric, biological, physical, and clinical information by morphing between clinical data and medical histories from cohorts of patients using machine learning. We anticipate that this perspective will shape the path toward introducing human heart simulations into precision medicine with the ultimate goals to facilitate clinical decision making, guide treatment planning, and accelerate device design.

## A brief history of cardiac modeling

The human heart beats 100,000 times a day, 40 million times a year, and three billion times throughout an average lifetime. Four centuries BC, Aristotle recognized the heart as the most important organ of our body and this observation still holds true today. In the early 17th century, William Harvey proved the function of the heart and the circulation of blood, a discovery that is considered the greatest medical achievement of all time. Throughout the past three decades, our understanding of the human heart has become much more quantitative, made possible by the close collaboration between medical scientists, biologists, and mathematical modelers. Today, we are at a critical turning point where we can confidently assume that, within the next decade, we will be able to model and simulate anybody’s individual personalized history of the heart. We take this opportunity to revisit what is possible to model and simulate today, what may become reality in the near future, and how mathematical modeling will guide medical device design and clinical decision making in the era of precision medicine.

### History of anatomic models

**Fig. 1:**
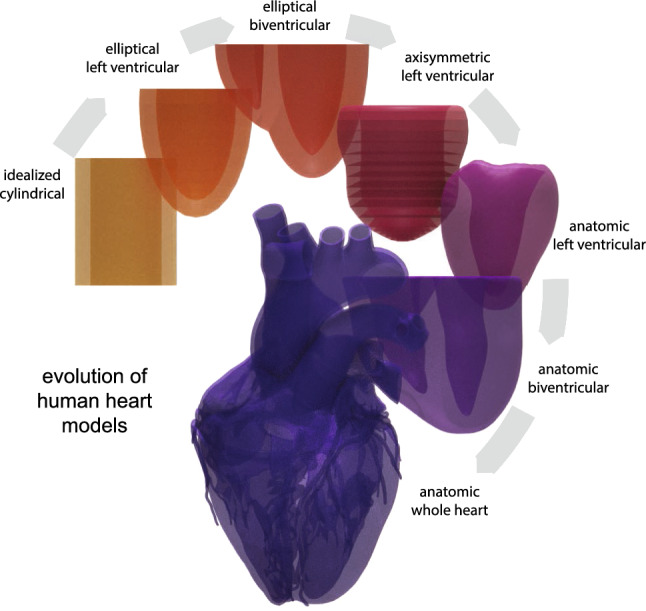
History of anatomic models. Increasing complexity of human heart models. Idealized cylindrical, elliptical left ventricular, and elliptical biventricular models allow for an analytical closed form expression for the fiber orientation. More realistic anatomic axisymmetric, left ventricular, biventricular, and whole heart models are based on real human heart geometries and fiber orientations

The art of mathematical modeling is to reduce a problem to the simplest possible mathematical description that is still manageable and, at the same time, detailed enough to capture the relevant effects. Figure 1 illustrates the art of human heart modeling through the hierarchy of models of increasing complexity. The first and simplest models of the heart are *idealized cylindrical models* that only focus on cross-sections of the left ventricle to rapidly develop and test constitutive models under physiological and pathological conditions (Guccione et al. 1991; Rodriguez et al. 1994). The next slightly more complex family of models are idealized *elliptical left ventricular models* with easy reproducibility and popular applications in benchmarking constitutive models (Nielsen et al. 1991; Eriksson et al. 2013; Land et al. 2015). The simplest models to study the interaction between both ventricles are idealized *elliptical biventricular models*, which can illustrate the effects of pulmonary and systemic hypertension, while still utilizing a generic geometry (Göktepe and Kuhl 2010). A major advantage of all three types of models is that their geometric simplicity provides an analytical representation of the fiber orientation, which is relevant to realistically model cardiac contraction, growth, and remodeling. The next more complex class of models, although geometrically simpler, are *axisymmetric left ventricular models* that can be constructed by rotationally sweeping a cross section of the left ventricular anterior free wall represented through a set of epi- and endocardial points with discrete fiber orientations (Guccione et al. 1995). More recent studies that focus on regional heterogeneities, for example introduced through prestrain, abandon the assumption of axisymmetry altogether and use full *anatomic left ventricular models* from personalized geometries (Genet et al. 2015). For electrophysiological simulations, where we are interested in extracting electrocardiograms under physiological and pathological conditions, we usually have to simulate both ventricles using *anatomic bi-ventricular models* (Sahli Costabal et al. 2016). Anatomic bi-ventricular models are also frequently used to simulate pressure-volume loops (Krishnamurthy et al. 2013) or to predict realistic excitation patterns in response to different treatment scenarios (Ramírez et al. 2020). The most complex models of the heart are undoubtedly *anatomic whole heart models* from real personalized geometries (Zygote 2014) that become unavoidable when studying the interaction between the atria and the ventricles, for example in all medical conditions that involve the valves. While enormous progress has been made since the first anatomic heart models in the early 1990s, creating personalized whole heart models, with all four chambers, the valves, the papillary muscles, and the chordae tendineae, with personalized excitation and contraction systems of Punkinje fibers and heart muscle fibers, and realistic in- and outflow conditions for the blood, remains one of the major challenges in bringing computational modeling closer to clinical use (Smith et al. 2011).

### History of physical models

Fortunately, not all simulations of the heart require complex whole heart modeling. The choice of the model depends strongly on the medical condition of interest, or, more precisely, on the relevant physics that govern this medical condition. Problems associated with the excitation of the heart, arrhythmias, or pacing, are represented through the integral equations of action potential propagation, in the simplest case through *monodomain models* (Aliev and Panfilov 1996). Monodomain models are usually sufficient for most excitation problems (Potse et al. 2006). Yet, there are specific conditions where differences in anisotropic conduction in the intra- and extracellular spaces are essential and *bidomain models* become necessary (Pathmanathan et al. 2010), for example when studying the transmembrane potential during unipolar stimulation or the magnetic field at the wave front (Luther et al. 2011). While electrical problems have historically been solved on a fixed grid using finite difference methods, more recent studies have adopted finite element methods with a view toward a monolithic coupling of excitation and contraction (Göktepe and Kuhl 2010). However, the electrical excitation problem typically requires a much finer resolution than the mechanical contraction problem, both in space and in time, and usually benefits from simple, high-resolution, regular grids. Problems associated with the contraction of the heart, heart failure, growth and remodeling, and pretty much all surgical procedures are represented through *mechanical models* characterized through the equations of motion or Newton’s second law (Nash and Hunter 2000). To study how cardiac excitation translates into mechanical contraction, it can be useful to adopt *electro-mechanical models* of excitation-contraction coupling (Cherubini et al. 2017; Quarteroni et al. 2017). For many practical applications, especially in the healthy heart, it can be sufficient to couple both phenomena weakly, first calculate the spatio-temporal excitation pattern, and then simulate the mechanical contraction. Under pathological conditions, however, mechano-electrical feedback and fully coupled solutions can become critical to correctly identify excitation wave trajectories, for example in ablation therapies (Sahli Costabal et al. 2017). Even more sophisticated *chemo-electro-mechanical models* trace the origin of the electrical excitation back to ionic currents across individual cells, for example, to understand the effects of drugs on the heart Sahli (Costabal et al. 2018). For many medical conditions, it is sufficient to model the heart as a deformable solid and represent the effects of the blood through transient pressure changes in both ventricles (Baillargeon et al. 2014). Yet, for conditions that are dominated by shear stresses, either across the endocardium or the heart valve leaflets, *computational fluid dynamics models* that represent details of the fluid flow become necessary (Taylor et al. 2013). However, fluid models alone are rarely used in cardiac simulations, mainly because the beating heart itself is a complex moving domain. This is why most recent studies focus on *fluid-structure interaction models* that capture not only the fluid flow within the contracting atria and ventricles, but also its interplay with the endocardium and the valves (Kaiser et al. 2020). Applications of fluid–structure interaction phenomena are abundant in cardiac medicine and range from surgical procedures (Mao et al. 2016), valve replacement (Ghosh et al. 2020), or valve repair (Rausch et al. 2017) to improved medical device design (Rotman et al. 2018).

### History of constitutive models

The beauty of most constitutive models of the heart is that they are hierarchical and modular in nature and easy to expand or combine with one another. For the electrical behavior, the simplest phenomenological model for excitable cells is the Fitz Hugh-Nagumo model (FitzHugh 1961; Nagumo et al. 1962) and its popular modification for cardiac cells, the Aliev-Panfilov model (Aliev and Panfilov 1996). More mechanistic ionic models represent the behavior of individual ion channels through transient gating variables and ionic currents (Karma 2013; Corrado and Niederer 2016; Fenton and Cherry 2008). The most popular model of this class for human ventricular cardiomyocytes is probably the ten Tusscher model ten (Tusscher et al. 2004) with several more recent modifications and a prominent benchmark study (Niederer et al. 2011). For the mechanical behavior, passive myocardial models have been developed for more than three decades, both isotropic and anisotropic (Guccione et al. 1991), and a recent benchmark study compares the different results (Land et al. 2015). In the spirit of modular models, depending on the type of application, numerous studies have added active behavior, either through active stress (Hunter et al. 1998) or active strain (Ambrosi et al. 2011; Göktepe et al. 2014; Rossi et al. 2012), prestrain ((Genet et al. 2015), and growth (Rodriguez et al. 1994) to the baseline passive elastic response to simulate realistic cardiac contraction and realistic pressure volume loops under physiological and pathological conditions. Interestingly, calibrating these models ex vivo (Sommer et al. 2015) versus in vivo (Genet et al. 2014) results in material parameter values that can differ by an order of magnitude or more (Aguado-Sierra et al. 2011; Rausch et al. 2013). This difference can obviously have massive implications when translating simulations into clinical practice (Chabiniok et al. 2016).

### What’s next?

Clearly, the ultimate objective of human heart modeling is the individualized prediction of different treatment outcomes with the goal to virtually select the most promising strategy within the paradigm of precision medicine (Taylor et al. 2013). Cardiac simulations are algorithmically challenging and computationally expensive; they naturally involve complex tasks such as mesh refinement, preconditioning, optimization, and parallelization (Hurtado and Rojas 2018; Jilberto and Hurtado 2018; Mei et al. 2018). With the rapid developments in machine learning, data-driven modeling, and physics-based simulation (Alber et al. 2019), we can now risk-stratify large patient groups and improve tailored cardiovascular therapies using machine learning strategies (Bom et al. 2019; Lyon et al. 2019). Intriguingly, precision medicine in cardiac health does not necessarily require a fully personalized, high-resolution whole heart model (Trayanova and Winslow 2011) with an entire personalized medical history (Gray and Pathmanathan 2018). Instead, precision human heart simulation can create a personalized model out of a population-based library with geometric, biological, physical, and clinical information (Segars et al. 2019), by morphing between real medical and clinical data from actual patients encoded in a finite number of fully reconstructed four-dimensional human heart models. This review will highlight first steps in this direction, not only as a purely academic exercise, but also as a translational path towards clinical decision making in full alignment with and endorsed by the regulatory agencies and guidelines.

## Electrophysiology—The healthy heart

**Fig. 2:**
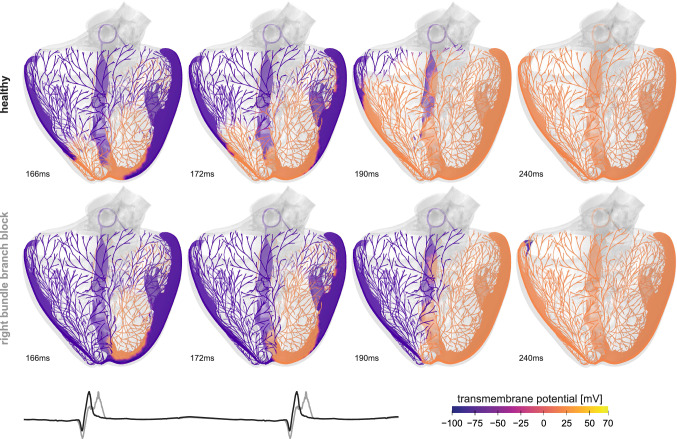
Electrophysiology—The healthy heart. Transmembrane potential across the left and right ventricles. Under physiological conditions, the Purkinje network excites the left and right ventricles simultaneously and induces synchronous ventricular contraction. Under pathological conditions of right bundle branch block, the left ventricle excites before the right ventricle and induces asynchronous contraction. The electrocardiogram highlights the differences between the physiological and pathological excitation pattern

### Motivation

In the USA alone, over 850,000 people die as a result of cardiovascular disease and more than 370,000 sudden cardiac deaths are related to arrhythmias (American Heart Association 2020). Arrhythmias are a leading risk factor for stroke which, in turn, is the leading cause of long-term disability and the second leading cause of death worldwide (World Health Organization 2018). Modeling cardiac excitation is critical to understand the origin of heart rhythm disorders and their treatment through anti-arrhythmic drugs, medical procedures, implantable devices, and surgical procedures (Sahli Costabal et al. 2020). In addition to the heart muscle itself, a critical component of the cardiac excitation system is the Purkinje network (Sahli Costabal et al. 2016). Composed of specialized fast-conducting cells, the Purkinje network is located in the subendocardium, right beneath the inner lining of the heart. Purkinje cells are larger than cardiomyocytes, with fewer myofibrils and more mitochondria. They are less contractile than cardiomyocytes; their main function is to conduct the excitation wave efficiently and more rapidly than any other cell in the heart. A functional Purkinje network is essential to create synchronized contractions of the left and right ventricles and maintain a consistent cardiac rhythm (Dubin 1996). The electric activation of our heart originates in the sinoatrial node located in the right atrium. From here, it spreads through the atria and reaches the atrioventricular node, the only electric connection between the atria and the ventricles. The bundle of His connects the atrioventricular node to the Purkinje network, which branches from the basal septum into the left and right ventricles. Although Purkinje fibers were first observed more than a century ago (Tawara 1906), there is still no in vivo imaging technique to fully reconstruct their geometry (Çetingül et al. 2011). This limitation has given rise to various methods to create model systems of the Purkinje network (Cherry and Fenton 2012) for both visualization purposes and computational simulations (Ijiri et al. 2008; Bordas et al. 2011; Sebastian et al. 2013). However, creating a Purkinje network on an irregular endocardial surface is challenging, but at the same time crucial for realistic physiological simulations.

### Simulation

We create the Purkinje network for rapid cardiac excitation as a random fractal tree (Sahli Costabal et al. 2016). This fractal tree grows in the left and right endocardial ventricular surfaces, starting from the left posterior and anterior fascicles and the right bundle branch. For the electrophysiology of the cardiac tissue, we adopt a bidomain model (Dal et al. 2012) with a modified Aliev Panfilov electrophysiology (Hurtado et al. 2016). We use the Living Heart Model (Baillargeon et al. 2014) as a baseline geometry and discretize the ventricles with 384,371 linear tetrahedral elements and 82,594 nodes. Within the heart, we create a Purkinje network with 1,868 branches and 1,046 terminals discretized by 10,757 line elements and 10,758 nodes. Here, instead of using a fixed-point iteration to solve the coupled problem of the myocardium and the Purkinje network (Landajuela et al. 2018), we directly connect the 1046 terminals of the Purkinje network to the endocardial surface using multi-point constraints (Abaqus 2020). We use Abaqus/Standard to simulate the heart throughout multiple cardiac cycles and initiate excitation by applying an external stimulus to the Purkinje network in the location of the atrioventricular node. From the simulation, we post-process the results to create a virtual electrocardiogram by placing electrodes in the left and right arms and the left leg. To compare healthy and diseased conditions, we simulate the baseline case and the condition of right bundle branch block for which we reduce the conduction velocity in the right bundle branch. This allows us to compare the healthy and diseased activation patterns, side by side, and identify the organ-level effects of local pathological alterations.

### Discussion

Figure 2 shows the importance of the Purkinje network in the electrical activity of the heart. This structure is key to predict a realistic activation sequence that will impact the mechanical response of the heart. We can see this in the electrocardiograms, which displays a sharp QRS complex, a feature that the simulation cannot capture without a fast conduction system. When including the Purkinje network, it is straightforward to simulate conduction pathologies, for example, right bundle branch block (Sahli Costabal et al. 2016). An accurate prediction of the heart’s electrophysiology under both physiological and pathological conditions is especially relevant when evaluating treatments such as cardiac ablation or resynchronization therapy, where it is not immediately clear which patients will benefit from the intervention (Strauss et al. 2011). A remaining challenge is the personalization of the Purkinje network. Since there is no method to image the Purkinje cells in a living heart, the identification of this structure relies on indirect electrical measurements including electroanatomical maps and electrocardiograms (Vergara et al. 2014). Truly personalized electrophysiology models would enable personalized treatment planning to address and prevent deadly arrhythmias and improve the mechanical performance of the heart (Prakosa et al. 2018). A typical example is ablation in atrial fibrillation, a procedure that selectively scars or destroys specific tissue regions to disrupt rhythm disorders in the upper chambers of the heart (Narayan et al. 2012). In a hybrid clinical-computational study of 15 patients with persistent atrial fibrillation, our personalized models found rotational activation, which was undetectable with conventional methods. Our findings suggest that computational modeling can identify non-local deflections to improve activation mapping and explain how and where ablation can terminate persistent atrial fibrillation (Sahli Costabal et al. 2018). Innovative technologies that enable real-time interactive simulations of cardiac electrophysiology (Kaboudian et al. 2019; Vasconcellos et al. 2020), for example based on physics-informed neural networks for cardiac activation mapping (Sahli Costabal et al. 2020), are an important step to translate these computational tools into clinical practice.

## Electrophysiology—Drug development

**Fig. 3:**
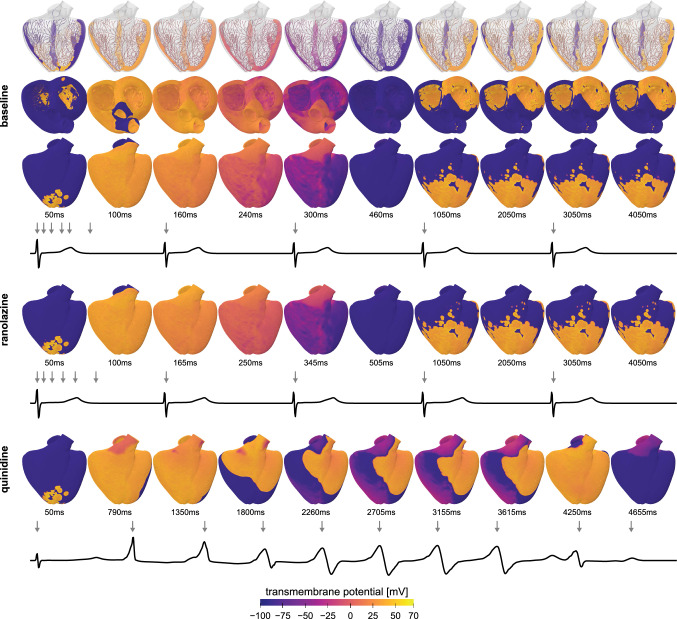
Electrophysiology—Drug development. Transmembrane potential profiles and electrocardiogram recordings under different pharmacological conditions: baseline condition, treatment with the low-risk drug ranolazine and the high-risk drug quinidine. Gray arrows in the electrocardiograms indicate the time points of the ten excitation profiles. Compared to baseline, treatment with ranolazine delays the repolarization period by 50 ms. For baseline and ranolazine, excitation is driven by the Purkinje network, with regular depolarization patterns recurring every second; quinidine triggers a sequence of rapid, irregular activation patterns

### Motivation

Drugs can induce changes in cardiac electrophysiology by interacting with specific ionic channels. Undesirable side effects of some of these compounds include cardiac arrhythmias. A particularly lethal type of arrhythmia is torsades de pointes (Dessertenne 1966), a condition associated with drugs that prolong the repolarization of the action potential (Po et al. 1999). Pro-arrhythmic risk evaluation is critical to avoid the introduction of potentially dangerous drugs to the market (Navarrete et al. 2013; Redfern et al. 2003). Yet, the high cost and long time needed to test new compounds hinders the discovery of new drugs. Current paradigms to evaluate cardiac safety are sensitive but not specific. This implies that they unnecessarily screen out potentially useful compounds. A recent paradigm shift to address this problem is the inclusion of high-fidelity computational models to quantify the effect of drugs in cardiac electrophysiology (Colatsky et al. 2016).

### Simulation

Multiscale modeling can help to characterize the effects of drugs in cardiac electrophysiology (Sahli Costabal et al. 2019). To compute the electrical activity under different pharmacological conditions, we adopt a monodomain model (Sahli Costabal et al. 2018) and simulate the excitation across the ventricular geometry of the Living Heart Model (Baillargeon et al. 2014). The model discriminates between four different cell types, Purkinje cells with 14 ionic currents and 20 internal variables to model the fast excitation (Stewart et al. 2009) and cardiomyocytes of the endocardium, midwall, and epicardium with 15 ionic currents and 39 internal variables (O’Hara et al. 2011). The anatomic bi-ventricular model consists of a regular mesh with cubic elements of size 0.3 mm, resulting in a mesh with 6,878,459 regular linear hexagonal finite elements, 7,519,918 nodes, and 268,259,901 internal variables. At the beginning of the simulation, we excite the Purkinje network in the location of the atrioventricular node, and use the automaticity of the Purkinje cells to drive the cardiac activation sequence for five seconds. We study the resulting activation sequence for three pharmacological conditions: baseline without drugs, and with the drugs ranolazine and quinidine. We simulate the effect of each drug at the cellular level by selectively blocking specific ionic currents using reported block-concentration measurements (Crumb et al. 2016). Ranolazine is a low-risk drug that selectively blocks the rapid delayed potassium rectifier current and the late component of the sodium current. Quinidine is a high-risk drug that blocks the rapid and slow delayed potassium rectifier currents and the transient outward potassium current (Colatsky et al. 2016).

### Discussion

Figure 3 illustrates how multi-scale modeling can provide insight into the emergence of drug-induced arrhythmias, from the subcellular scale of individual ion channels to the organ scale of the entire heart. The simulation predicts slightly altered activation patterns with prolonged QT intervals for the low risk-drug ranolazine and the spontaneous development of torsades de pointes for the high-risk drug quinidine. These multi-scale simulations correlate mechanistically what a pharmacologist sees in a single cell action potential to what a physician sees in a clinical electrocardiogram. They allow us to quantify the interaction between specific compounds and ionic currents at the cellular scale and compute the overall response in terms of global activation profiles and electrocardiograms at the whole organ scale. We validated this approach experimentally by analyzing isolated cardiomyocytes exposed to the drugs dofetilide and nifedipine at different concentrations, predicting the whole heart response, and comparing the prediction against electrocardiograms of Langendorff perfused heart preparations exposed to both drugs (Sahli-Costabal et al. 2020). A major challenges of this mechanistic multiscale approach is the high computational cost to evaluate a single drug at a single concentration. We have recently embedded this model into a physics-based machine learning framework (Alber et al. 2019) to reduce the computational cost and predict the risk categories of 22 drugs (Sahli-Costabal et al. 2020). We have successfully created surrogate models that combine the high-fidelity three-dimensional simulations with low-fidelity one-dimensional strands of cells and used this multi-fidelity approach for uncertainty quantification (Mirams et al. 2020) and sensitivity analysis (Sahli Costabal et al. 2019). Recently, we have extended this technique towards a classification setting to detect the presence or absence of arrhythmias (Sahli Costabal et al. 2020). On a fundamental level, this approach provides mechanistic insights that can help researchers, pharmaceutic companies, and regulatory agencies to accelerate drug development and design effective and safe drugs. With a view towards precision medicine, our technology could be used for personalized drug safety evaluation (Margara et al. 2020). To incorporate population variability, we simply need to know the individual cellular electrophysiology of different patients or patient populations, and can then predict their individual arrhythmic risk. An important immediate application would be sex-specific drug safety evaluation.

## Cardiac mechanics—The healthy heart

**Fig. 4:**
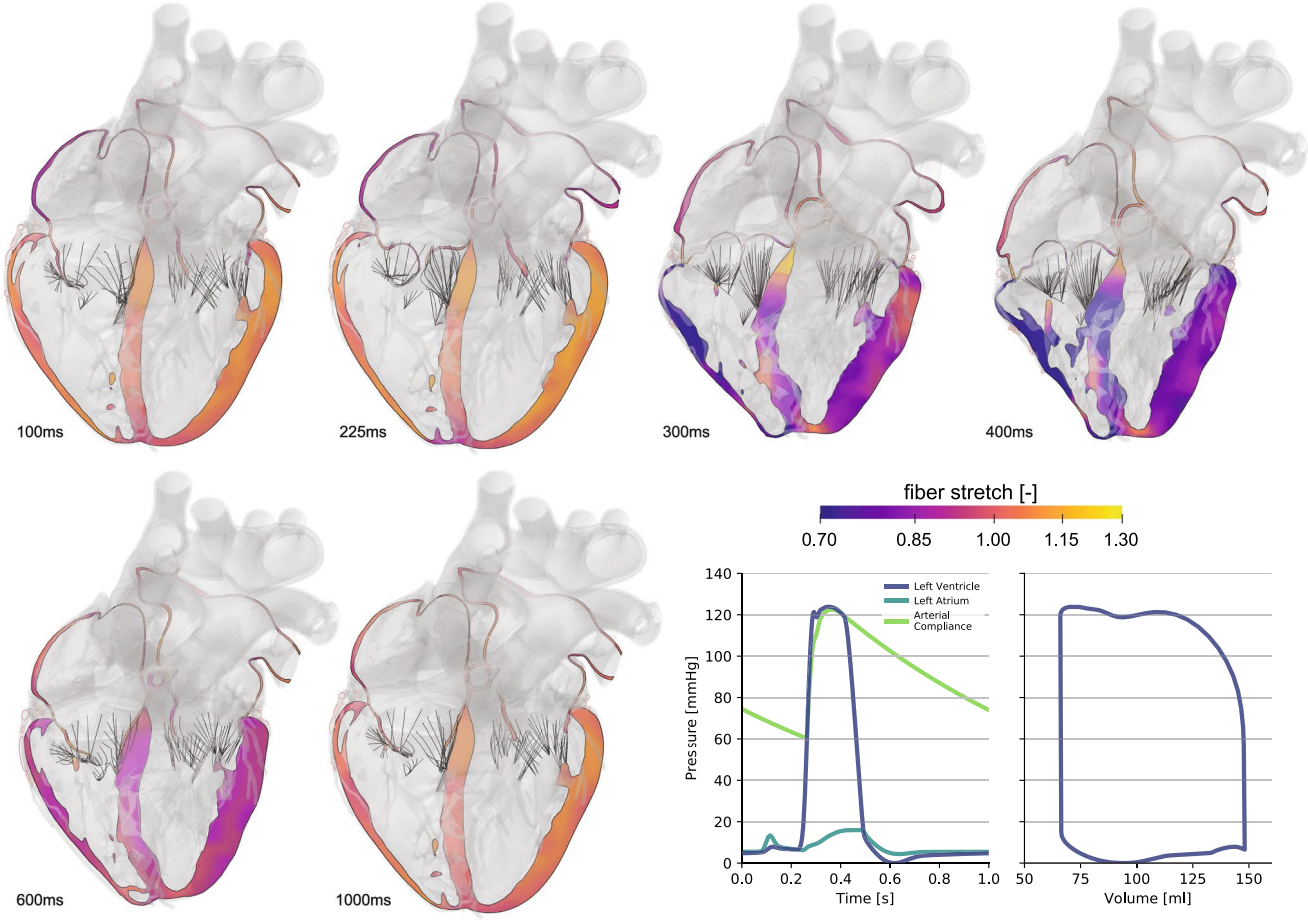
Cardiac mechanics—The healthy heart. Deformed configuration with fiber stretch profiles throughout the cardiac cycle. The long-axis view illustrates the dynamically changing fiber stretch during active contraction and passive filling. The graphs summarize the temporal inter-chamber pressure evolution for the left side of the heart, and the pressure-volume loop for the left ventricle

### Motivation

The main function of the heart is to pump blood through the cardiovascular system to continuously supply all other organs with oxygen and nutrients while removing carbon dioxide and other waste products. Upon electrical depolarization, the muscle cells in the heart release calcium ions, which activate actin-myosin interaction, sarcomere shortening, and active muscle contraction. An in-depth biophysical insight into the ventricular mechanics through multi-scale computational modeling requires realistic constitutive models that characterize this nonlinear, anisotropic, passive, and active tissue behavior, as well as the in vivo geometry, microstructural architecture, hemodynamic loading, and kinematic constraints of the heart. With a view toward personalized simulations, we need to calibrate all model parameters, loads, initial conditions, and boundary conditions to accurately match the physiological response.

### Simulation

Figure 4 illustrates an anatomically accurate, whole heart model of a human heart (Baillargeon et al. 2014) created from high-resolution magnetic resonance images of a healthy, 50th percentile U.S. male (Zygote 2014). Data acquisition and image reconstruction were performed at 70% diastole from 0.75-mm-thick slices using a medium soft-tissue kernel with retrospective electrocardiogram gating. The model includes all four chambers, the tricuspid, mitral, pulmonary, and aortic valves, and the chordae tendinae. It also includes the major vessels, the aorta, the pulmonary arteries, and the superior vena cava, together with the coronary arteries and veins and some cardiac fat tissue. The muscle fiber orientation follows rule-based algorithms motivated by histological observations and diffusion tensor magnetic resonance images (Lombaert et al. 2012; Bayer et al. 2012). To simulate the dynamic response of the heart, we adopt the theory of finite elasticity and characterize the mechanical behavior of the heart using the conservation laws of mass, linear momentum, and angular momentum. In addition, we introduce constitutive equations that define the relation between stress and strain, active contraction, prestrain, growth, and remodeling. We adopt a quasi-static approach and postulate that inertia and damping effects are negligible (Baillargeon et al. 2014). To characterize the response during passive filling, we adopt an orthotropic invariant-based constitutive model (Holzapfel and Ogden 2009; Propp et al. 2020). To describe the response during active contraction in the ventricles, the atria, and the chordae tendineae, we adopt the concept of time-varying elastance (Walker et al. 2005) and introduce the active stress as a function of the regional action potential and the cardiomyocyte stretch according to Frank-Starling’s law (Peirlinck et al. 2019). For simplicity, instead of embedding the heart in the pericardium through spring-type boundary conditions (Pfaller et al. 2019), we constrain the heart kinematically through homogeneous Dirichlet boundary conditions at the outlets of the proximal vasculature (Peirlinck et al. 2019). To characterize the hemodynamic boundary conditions, we couple the beating heart model to a lumped-parameter representation of the arterial and venous systemic and pulmonary circulations. This coupling allows for a closed-loop characterization of the cardiovascular flow through unidirectional fluid exchanges driven by pressure gradients which, in turn, are a result of the mechanically contraction of the heart (Baillargeon et al. 2014). Within this computational framework that describes the interaction between the beating heart and the circulatory system, we calibrate the constitutive parameters for the ventricles, the atria, the valves, and the proximal vasculature to match the experimentally measured stiffnesses and global clinical metrics including the ejection fraction and the left ventricular twist (Peirlinck et al. 2019). Specifically, from magnetic resonance images of the left ventricle of five normal human subjects, we extract left ventricular volumes and compare them against strain measurements from tagged magnetic resonance imaging to identify the passive and active material parameters (Genet et al. 2014). Since the geometry of the heart is constructed at 70% diastole with the heart already hemodynamically loaded, we estimate the in vivo stress state at the beginning of the simulation using an inverse method (Gee et al. 2010; Peirlinck et al. 2018). We simulate five consecutive cardiac cycles during which we initiate contraction using the electrical activation sequence from Sect. 2. After three cycles, the heart reaches a cyclic steady-state equilibrium.

### Discussion

Figure 4 illustrates the overall deformation of the heart and the evolution of the fiber stretch throughout a cardiac cycle. The graphs summarize the pressure evolution in time and the pressure-volume relationship. The simulated volumes ranging from 69-148ml and the pressures up to 122mmHg agree well with clinical observations (Klingensmith 2008). Our simulated ejection fraction of 56% agrees well with the physiological ranges of 50-65% (Phibbs 2007). Our maximum left-ventricular apex-base shortening of 13.4 mm is well in the range of commonly reported values of 11.2 ± 3.8 mm (Rogers et al. 1991) and the left ventricular twist of $10.9^{\circ }$  agrees with reported ranges of $7.7 \pm 3.5^{\circ }$ (Takeuchi et al. 2006). This suggests that we can confidently and robustly model the physiology of the healthy beating human heart. In a next step, we can use this simulation tool to probe pathological conditions and guide device design and treatment planning in cardiac diseases such as stenosis (Wisneski et al. 2020), regurgitation, ischemia (Sack et al. 2020), or heart failure (Peirlinck et al. 2019). With a view towards precision medicine, it would be relatively straightforward, although probably time intensive, to personalize the heart geometry from individual magnetic resonance images. It would be more complex, but possible, to personalize simple sets of material parameters (Genet et al. 2014). While personalizing more complex features like fiber directions, prestrain, or growth might not be entirely possible non-invasively with the available acquisition techniques today, it remains questionable whether this level of personalization is really necessary to improve treatment planning.

## Cardiac mechanics—Pacing lead failure

**Fig. 5:**
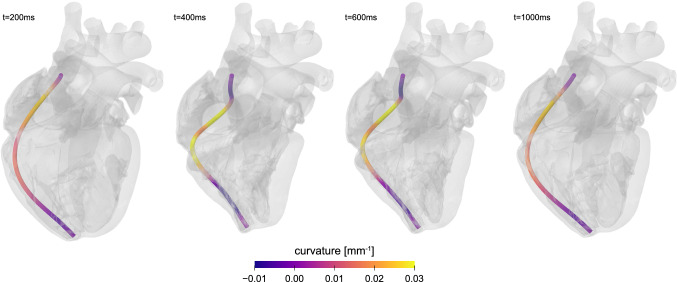
Cardiac mechanics—Pacing lead failure. Curvature changes along the pacing wire throughout the cardiac cycle. Large curvature values and large curvature changes are an indicator for pacing lead failure. The curvature alternates between positive and negative values along the wire. It takes maximum values of 0.03 $\hbox {mm}^{-1}$ in the upper third of the wire during the phase of maximum contraction

### Motivation

For patients who suffer from arrhythmias, an irregular or abnormal beating of the heart, implantable cardioverter defibrillators and artificial pacemakers continue to be life-saving devices. While implantable cardioverter defibrillators treat fast or chaotic rhythm disorders (Luther et al. 2011), pacemakers generally correct heart beats that are too slow or out of synch. They require a battery-powered unit that is placed in the chest, underneath the skin, where it is easily accessible for battery replacement (Kotsakou et al. 2015). Pacemaker leads are electrodes that connect the device to the regions within the heart that require the additional stimulus. As such, they are permanent implants that are expected to perform for many years, in an environment that contracts 100,000 times a day, without failure (Trohman et al. 2004). Naturally, the durability of the leads is of critical importance. Current research seeks to design soft devices with properties similar to the surrounding tissues to optimize seamless integration and minimize damage (Sim et al. 2020). Mechanical damage can result in pacing lead failure and the loss of electrical function (Mulpuru et al. 2017). However, it is difficult to measure the deformations and forces of the pacemaker leads in vivo. Simulating pacemaker leads in the living heart provides unique insight into the mechanical deformation of the electrodes throughout the cardiac cycle (Zhou et al. 2017). Knowing this mechanical deformation is critical to access the long-term durability of the implant. The noninvasive nature of this assessment provides a physiologically accurate method to test new and existing devices without exposing the patient to unnecessary risk.

### Simulation

The pacemaker lead is a long flexible wire of a length on the order of 150 mm. In a finite element setting, we can represent it with beam elements. It has a cross sectional area of 3.0 mm, which is magnified in Fig. 5 for illustrative purposes. The lead’s moment of inertia is 0.5 $\hbox {mm}^4$, the axial stiffness is 9 N/mm, the bending stiffness is 17 $\hbox {Nmm}^2$, and the torsional stiffness is 30 Nmm/rad. To simulate the mechanical deformation that the pacemaker lead experiences during the cardiac cycle, we adopt the Living Heart model from Sect. 4. The simulation uses Abaqus/Explicit (Abaqus 2020) and begins with filling the heart with blood up to 70% of the diastolic phase. During this process, which lasts 0.3 seconds, we virtually insert the pacemaker from the right atrium into the right ventricle through the superior vena cava. To facilitate the positioning of the pacemaker lead, we introduce an axial connector element between the distal end of the lead and the epicardial surface of the right ventricular apex. This location marks the region of the heart where the electrical stimulus is needed. We guide the placement through a rigid tubular structure which ensures that the lead follows a natural insertion path. By shortening the connector element to a final length close to 0 mm, we effectively pull the pacemaker lead tip from the superior vena cava to the right ventricular apex. Figure 5 illustrates the placement of the pacemaker lead within the beating heart. Once the insertion process is complete, we fix the distal end of the lead to the epicardial surface and pin the proximal end. We then simulate three consecutive cardiac cycles to establish a cyclic steady-state equilibrium. During these three beats, we model the interaction between the pacemaker lead and the heart using a general contact approach. Specifically, we specify contact between the pacemaker lead and the epicardial surfaces of the right atrium and ventricle. In addition, we specify contact between the pacemaker lead tip and the pacemaker lead path structure to ensure the proper final location of the tip.

### Discussion

A pacemaker lead is a thin flexible wire. During the cardiac cycle, its main mode of mechanical deformation is bending. The pacemaker lead curvature is the primary kinematic quantity of interest to quantify this deformation mode. We can characterize the curvature through the section curvature, the curvature change about two orthogonal planes. Figure 5 illustrates the curvature along the lead at various time points throughout the cardiac cycle. The curvature increases as the heart contracts. It takes maximum values of 0.03 $\hbox {mm}^{-1}$ in the upper third of the pacing wire. This corresponds to bending the wire around a sphere with a minimum radius of 3.3 mm, about one third of the dimensions of the heart. This agrees well with the deformation of the wire at 400 ms and 600 ms, in the middle two images of Fig. 5, during which the heart is contracting. At 200 ms and 1000 ms, when the heart is relaxing, the curvature in the same region decreases to 0.015 $\hbox {mm}^{-1}$, corresponding to a sphere with a radius of 6.6 mm, roughly on the order of the width of the heart. Simulating the deformation of the wire allows us to visualize how the curvature, and with it the stress in the wire, changes along the length and throughout the cardiac cycle. It allows us to virtually probe how the wire deformation would change upon changing the wire length, diameter, stiffness, or material. With a view towards precision medicine, it would be straightforward to personalize the final pacemaker lead location, the length of the lead, and the dimensions of the heart. A better understanding of pacing wire deformations is critical to reduce high cycle fatigue, prevent pacing lead failure, and improve wire durability with the ultimate goal to improve the lifetime of pacemaker leads and reduce the need for replacement surgery.

## Cardiac mechanics—Heart failure

**Fig. 6:**
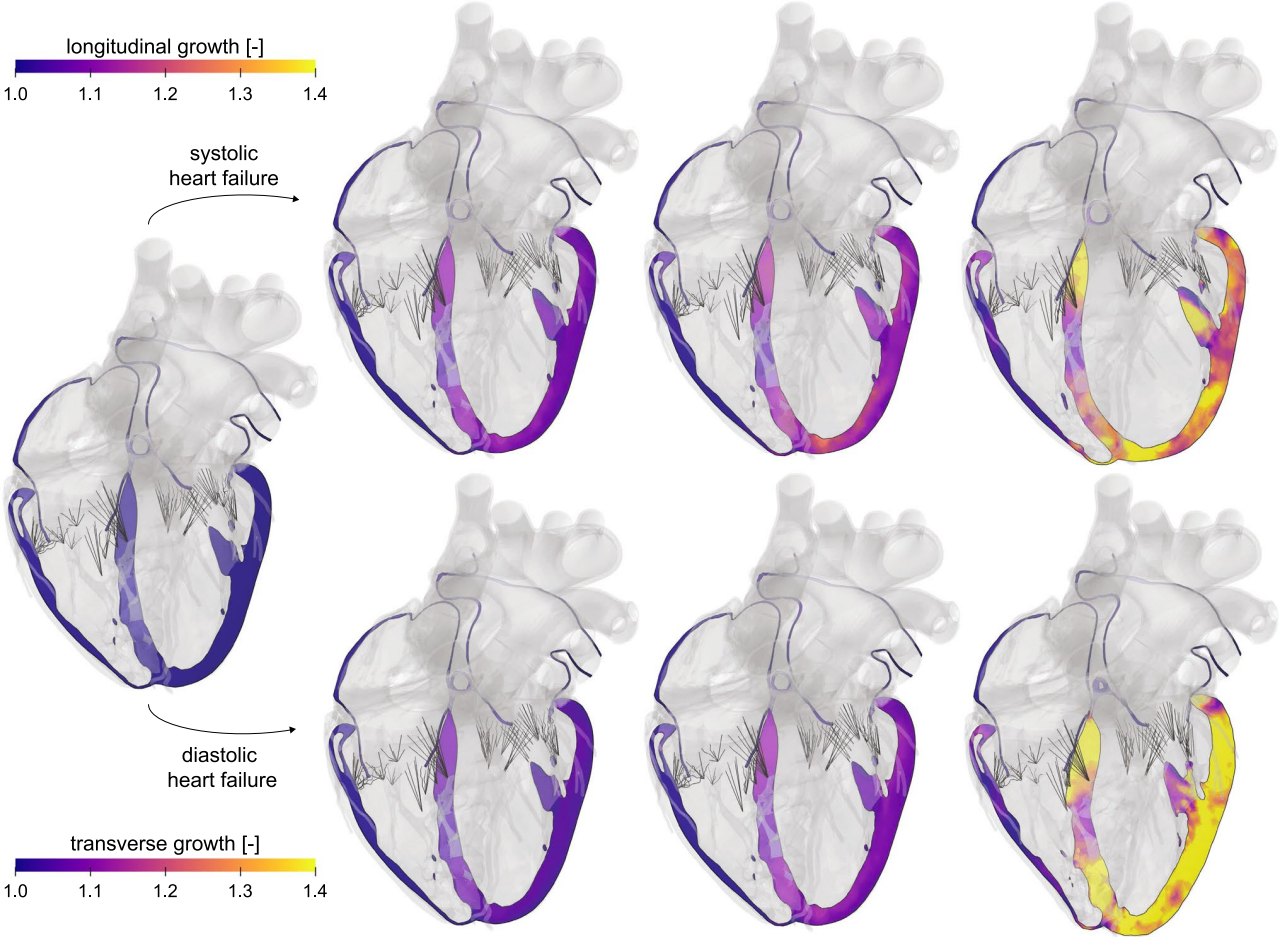
Cardiac mechanics—Heart failure. Longitudinal and transverse growth during systolic and diastolic heart failure. Systolic heart failure is associated with eccentric hypertrophy, a serial addition of sarcomeres, an increase in volume, and loss of ellipticity. Diastolic heart failure is associated with concentric hypertrophy, a parallel addition of sarcomeres, an increase in wall thickness, and a preservation of ellipticity. The color codes visualize the relative lengthening and thickening of the heart muscle cells

### Motivation

Heart failure is a progressive chronic condition in which the heart is unable to pump enough blood to meet the body’s oxygen demand. With a five-year mortality rate of 50%, heart failure remains–and will remain in the foreseeable future–one of the most common, costly, disabling, and deadly medical conditions worldwide (World Health Organization 2017). Most cases of heart failure result from adverse cardiac growth and remodeling in response to increased hemodynamic loading (Ambrosi et al. 2019; Göktepe et al. 2010), for example, provoked by the leakage or stenosis of one of the valves or by myocardial infarction (Saez and Kuhl 2016). In many cases, cardiac growth and remodeling is a compensatory, useful, and protective mechanism to restore homeostatic equilibrium (Cyron and Humphrey 2017). It some cases, however, it can become non-compensatory and initiate negative feedback mechanisms (Grossman 1980). The underlying structural response to cardiac growth and remodeling manifests itself in two major patterns: eccentric hypertrophy associated with myocyte lengthening caused by a chronic overload in volume and concentric hypertrophy associated with myocyte thickening caused by a chronic overload in pressure (Niestrawska et al. 2020). In both cases, these mechanisms lead to pathological structural alterations that compromise the heart’s electrical and mechanical function (Sahli Costabal et al. 2017). Despite recent advancements of pharmaceutical, surgical, device, and tissue engineered therapeutic strategies, heart-failure-induced morbidity and mortality rates remain high (Udelson and Stevenson 2016). One of the most pertinent clinical questions in treatment planning is to anticipate and predict the rate of disease progression (Witzenburg and Holmes 2017). Cardiac growth models have the potential to provide mechanistic insight in disease onset and progression (Rausch et al. 2017) and guide clinical decision-making or the design of emerging therapies (Chabiniok et al. 2016). However, since disease progression is highly sensitive to the personal history, and with it, personal model parameters (Kassab 2009), the timeline of heart failure varies significantly among affected individuals. This suggests that, ideally, calibrating cardiac growth models and quantifying growth and remodeling propensity should be done on an individual, personalized basis (Peirlinck et al. 2019).

### Simulation

Cardiac growth models typically build on the theory of finite growth (Rodriguez et al. 1994), which postulates a multiplicative decomposition of the deformation gradient into an elastic tensor and a growth tensor (Göktepe et al. 2010). Here, we assume that the growth tensor is transversely isotropic with respect to the cardiomyocyte direction and can be parameterized in terms of scalar longitudinal and transverse growth multipliers. These growth multipliers represent the serial deposition of new sarcomeres and the parallel deposition of new myofibrils. Within the finite element setting (Abaqus 2020), we represent the growth multipliers as internal variables and store and update the current growth state locally, on the integration point level, at each point in time. We assume stretch-driven growth kinetics for both longitudinal and transverse growth (Kerckhoffs et al. 2012). We simulate the timeline of these two pathologies within the Living Heart (Baillargeon et al. 2014) through a combination of eccentric and concentric hypertrophy triggered by left ventricular overload. To model the physiological end-diastolic state and the homeostatic local stretch state, we apply a left ventricular and atrial pressure of 4 mmHg and a right ventricular and atrial pressure of 2 mmHg. To model systemic overload, we double the left ventricular and atrial pressures to 8 mmHg while keeping the right ventricular and atrial pressures at their baseline value of 2mmHg. We gradually increase the pressure, keep it at its maximum value to allow the ventricles to grow, and then unload the heart to explore the effects of eccentric or concentric hypertrophy Genet et al. (2016).

### Discussion

Our results in Fig. 6 not only agree qualitatively with the primary effects of heart failure (Göktepe et al. 2010)–ventricular dilation in systolic heart failure and wall thickening in diastolic heart failure–but also allow us to predict characteristic secondary effects of papillary muscle dislocation, mitral annular dilation, regurgitant flow, and outflow obstruction (Rausch et al. 2017). These results agree favorably with clinical observations in patients with systolic and diastolic heart failure. In contrast to previous macroscopic growth models that prescribe a phenomenological rule to drive the remodeling process (Rodriguez et al. 1994), these personalized models induce growth as a natural consequence of overload, which is self-regulated and converges toward a homeostatic equilibrium state. This implies that the emerging growth pattern is heterogeneous, and naturally incorporates the regionally varying response under personalized baseline conditions as the homeostatic state. Implementing this framework in a four-chamber whole heart model allows us to couple primary geometric changes to secondary effects that drive changing in-and out-flow conditions and enable predicting disease progression with its characteristic negative and positive feedback loops (Genet et al. 2016). Upon appropriate calibration, the cardiac growth models have the potential to link cell level characteristics, e.g., an increase in the serial sarcomere number, to whole organ form and function, e.g., an increase in end-diastolic volume and a decrease in ejection fraction, with the ultimate goal to estimate the risk of heart failure and support decision making on an individualized, personalized basis. We have prototyped this correlation by combining hierarchical modeling, Bayesian inference, and Gaussian process regression using an eight-week long study of volume overload in six pigs. By correlating overload-induced alterations on the subcellular, cellular, and organ scales we found that the serial sarcomere number explained 88% of cardiomyocyte lengthening, which, in turn, explained 54% of cardiac dilation (Sahli Costabal et al. 2019). However, stretch-driven growth is only one of the many proposed mechanobiological stimuli that regulate cardiac hypertrophy (Opie et al. 2006). To calibrate and validate the stretch-driven growth model, we analyzed chronic heart failure in pigs using subject-specific growth models and physics-based machine learning. Our study revealed that stretch-driven growth alone explained 52.7% of the observed changes in cardiomyocyte morphology (Peirlinck et al. 2019). With a view toward precision medicine, studies like these are vital to reveal the intersubject variability of growth, which emphasizes the importance of personalized growth parameters to accurately predict the timeline of heart failure for an individual patient. Knowing the individual timeline of a failing heart is critical for personalized treatment planning, as shown in Sect. 7, 8, and 9 and on ventricular assist devices, edge-to-edge repair, and annuloplasty.

## Cardiac mechanics—Ventricular assist devices

**Fig. 7:**
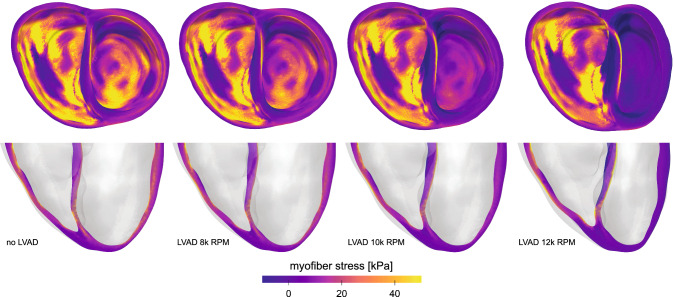
Cardiac mechanics—Ventricular assist devices. Free wall and septal stress distribution in dilated cardiomyopathy without treatment, left, and with a left ventricular assist device with increasing rotational speed from 8000 to 12,000 revolutions per minute, from left to right. The deformed geometries and the myocardial stresses reveal the increasing impact of device support with increasing rotational speed

### Motivation

A ventricular assist device is a battery-operated, mechanical pump that is used for end-stage heart failure patients to help their left ventricle pump blood to the rest of the body (Rose et al. 2001). In recent years, left ventricular assist devices have become increasingly popular as more than just a bridge-to-transplant therapy. The high incidence of right ventricular failure following left ventricular assistance reflects an undesired consequence of treatment, which has been hypothesized to be related to the mechanical interdependence between the two ventricles (Kavarana et al. 2002; Dang et al. 2006; Maeder et al. 2009). Although the potential implications of ventricular interactions on right ventricular function during left ventricular assist device support are well-appreciated, no study has yet proven, in any setting, that left ventricular unloading and septal shift can lead to right ventricular failure. This is because it is physically impossible to separate the hemodynamic effects of the serial and parallel contributions of right-left ventricular interactions in a patient or even in experimental preclinical studies. Predictive computation models offer the potential to uncover the mechanisms of treatments whose actions cannot be easily determined by experimental or imaging techniques (Kerckhoffs et al. 2007; Baillargeon et al. 2014). Computational modeling is well-suited to investigate and elucidate the individual contributions of hemodynamic factors and explore left ventricular assist device complications. However, research efforts have been impeded by the substantial complexities involved in coupling a simulated circulatory system with geometrically realistic models of the heart. Only recently have computational models had the necessary sophistication to model this coupled behavior (Lim et al. 2010; Sack et al. 2016). Consequently, very limited research has been undertaken to explore the effect of left ventricular assist device function on ventricular mechanics (Sack et al. 2018), and no study has investigated the important issue of right heart failure.

### Simulation

We previously created a model of a failing left ventricle supported with partial left ventricular assistance in a four-chamber generic heart model (Sack et al. 2016). We then modified this representation to include a biventricular model of a patient with dilated cardiomyopathy (Sack et al. 2018) using an explicit, dynamic, mechanical simulation with $\sim 80,000$ ten-noded tetrahedral elements (Abaqus 2020). We simulate left ventricular assist device therapy using realistic pressure-flow relations of a commonly used left ventricular assist device to capture assisted flow for device operation over a broad range of rotational speeds. By analyzing the resulting changes in left ventricular pressure generation, total blood flow, myocardial stress, and septal wall motion, we quantify the relative influences of these factors on right ventricular function (Sack et al. 2018). The model of chronic heart failure without left ventricular assist device support represents a critical patient with advanced heart failure (Solomon et al. 2011). The left ventricle is substantially overloaded with an end-diastolic volume of 254 ml, an end-diastolic pressure of 23 mmHg, and a left ventricular ejection fraction of 12%. The support through the left ventricular assist device improves these functional metrics: The diastolic loading of the left ventricle decreases and the left ventricular ejection fraction increases with increasing rotational speed of the device. Figure 7 illustrates the overall geometry and the myofiber stress distributions at end diastole to showcase stresses and the deformed configuration at maximum volume loading. The four images contrast the dilated cardiomyopathy state without treatment and with implanted left ventricular assist device operating at rotational speeds of 8000, 10,000, and 12,000 revolutions per minute. The resulting stress distributions reveal geometrically relevant stress characteristics that evolve with increased left ventricular assist device operation. Interestingly, the large stresses in the left ventricular endocardium caused by volumetric loading at end diastole decrease with left ventricular assist device support and appear to dissipate with maximum left ventricular assist device operation of 12,000 revolutions per minute. However, a localized region of tensile myofiber stresses appears and grows with increased assist device support on the left ventricular side of the septal wall near the base. In addition, the assist device promotes a localized region of compressive myofiber stresses on the right ventricular side of the septal wall.

### Discussion

Computational simulations provide a window into the effects of left ventricular assist devices on myocardial dynamics. The contour plots of the free wall and septal stresses illustrate the potential of computational modeling to quantitatively compare the myofiber stress in the ventricular wall for varying rotational speeds of the assist device. Specifically, we have created a geometrically and physically realistic model of an end-stage failing heart with representative systolic and diastolic myocardial material properties coupled to lumped parameter Windkessel-like models of the pulmonary and systemic circulations (Sack et al. 2018). This allows us to study cardiac mechanics and dynamics under realistic loading conditions, including preload and afterload in both ventricles. The simulation successfully reproduces the effects of left ventricular assist device support and can be personalized by apply individualized pressure-flow characteristics of any commonly used device. The present simulation represents a significant improvement over previous modeling efforts (Sack et al. 2016) in that it precisely quantifies the effects of a left ventricular assist device on a chronically rather than acutely failing heart, based on a personalized anatomically accurate biventricular model and device-specific pressure-flow characteristics, rather than constant flow rates. The study shows that left ventricular assist device support significantly reduces the stress in the left ventricular wall and, to a lesser extent, the stress in the septal wall (Sack et al. 2018). Unexpectedly, these improvements induce secondary negative effects on the right ventricle, which experiences a rightward shift toward higher end-diastolic pressures and larger end-diastolic volumes with left ventricular assist device support. This keeps the right ventricular stresses high. Additionally, we observed potentially negative effects on the interventricular septum. Left ventricular assist device support introduces an unnatural bending of the septum, which results in increased localized myofiber stresses. Such deformations are similar to those of a plate subjected to bending. As Fig. 7 reveals, this induces device-speed-dependent regions of tensile stress on the left ventricular side and regions of compressive stress on the right ventricular side of the septal wall. Chronically elevated stresses are critical as they can modulate important myocardial properties including gene expression, molecular makeup, structure, and function. It remains unknown to which extent these abnormal stresses in the myocardium or septum have implications for myocardial function. With a view towards precision medicine, it is critical to personalize the biventricular geometry to reflect the patient’s individual disease state and personalize the optimal rotational speed of the assist device to strike the right balance between supporting the heart and, at the same time, reducing device-induced wall stresses and septal bending.

## Cardiac mechanics—Edge-to-edge repair

**Fig. 8:**
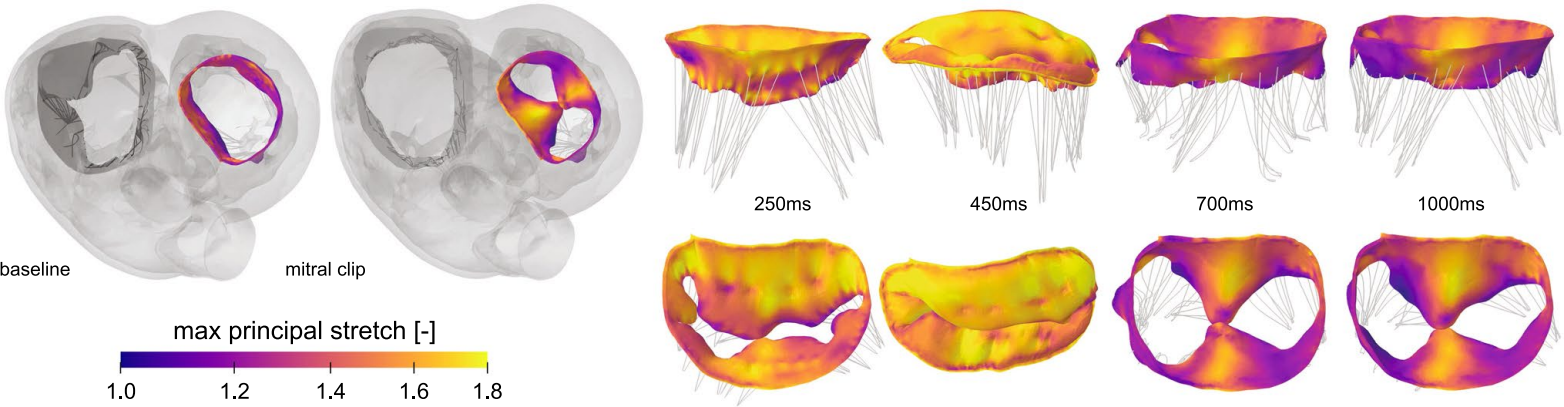
Cardiac mechanics—Edge-to-edge repair. Maximum principal stretches across the mitral leaflets at multiple time points throughout the cardiac cycle. Baseline model and degenerative mitral regurgitation model, where the mitral clip is simulated using a connector element between nodes on each leaflet, left, and maximum principal stretch in side and top views, right

### Motivation

Understanding the in vivo loading and boundary conditions for cardiovascular implants remains a major challenge for device design manufacturers. Characterizing these conditions is not only critical to guide the design specifications for cardiac implants but also to select the optimal device and device placement for each individual patient. Typically, device design and placement are studied using benchtop cadaveric tests, animal studies, and clinical imaging. The precise quantification of the forces between the device and the tissue is virtually impossible, especially in the beating heart. Mitral regurgitation is a chronic condition of the left heart during which the mitral valve does not close completely in the systole and allows leakage of blood back to the left atrium. Edge-to-edge mitral valve leaflet approximation, the so-called Alfieri procedure, is often performed for patients with ischemic mitral regurgitation (Fucci et al. 1995). Because of the success of this procedure, transcatheter devices to perform leaflet approximation are gaining increasing attention. Understanding the loads these implants have to endure under physiological conditions is critical to develop an effective and long-lasting device. In developing these implants, the Alfieri stitch tension forms an important design specification. Traditionally, this tension is measured using animal models (Nielsen et al. 2001; Timek et al. 2004) and benchtop simulators (Jimenez et al. 2006). Here we illustrate an alternative approach that uses a computational human heart model to estimate the forces on the mitral valve leaflet (Baillargeon et al. 2014, 2015). Concomitantly, this approach allows us to non-invasively investigate the effect of physiological parameters and dimensional changes associated with structural heart disease including systolic blood pressure, left ventricular dimension, and annular dilation.

### Simulation

We use the Living Heart Model (Baillargeon et al. 2014) to evaluate the leaflet approximation force $F_A$. We create a degenerative mitral regurgitation model by disconnecting several marginal chordae from the anterior leaflet. We use a connector element, a discrete structural element in Abaqus/Explicit, to prescribe specific kinematic behavior between two nodes of the model (Abaqus 2020). When placed between two nodes in the A2-P2 regions of both mitral leaflets, the connector element forms a simplified representation of a leaflet approximation device. We approximate the leaflet anatomy by prescribing the final length of the connector and represent the device’s compliance by assigning an elastic stiffness to the connector element. The force in the connector element throughout the entire cardiac cycle serves as an estimate of the approximation force $F_A$ during both systole and diastole.

### Discussion

Figure 8 illustrates the deformation and the maximum principal stretches of the leaflets at multiple time points throughout the cardiac cycle. The stretch distribution during diastole indicates the location of elevated stresses around the A2-P2 region as a result of leaflet approximation. From the connector element, can directly extract the simulated leaflet approximation force $F_A$ and compare it to experimentally measured forces from animal studies. The simulated approximation force $F_A$ directly depends on the selected connector stiffness that represents the stiffness of the device. For a compliant device, our simulation estimates a peak diastolic force of 0.22 N, while an entirely rigid device results in a peak diastolic stitch tension of up to 0.31 N. These results are in good agreement with animal studies, where the peak diastolic force ranges from 0.26 N (Nielsen et al. 2001) to 0.28 N (Timek et al. 2004). In addition, the simulations reveal a second peak during the systolic part of the cardiac cycle, whereas the animal studies only report a single peak during diastole. The systolic peak of the simulation occurs during isovolumetric contraction. This can be attributed to several different factors including device placement, i.e., location of the connector, anatomical artifacts, and device behavior. While experimental studies to understand the effect of these factors on the approximation forces are not straightforward to perform, computational simulations can easily screen the landscape of different process parameters. By using personalized geometries and a personalized representation of the current regurgitation state, we can optimize the device and its location for each individual patient. Using personalized physics-based simulations to augment benchtop and animal studies is critical to understand the interaction between cardiac implants and anatomy. Simulations can provide insight into the in vivo device mechanics, guide the design of efficient cardiovascular implants, and optimize treatment on a personalized basis.

## Cardiac mechanics—Annuloplasty

### Motivation

In the healthy heart, the mitral valve manages unidirectional blood flow from the left atrium to the left ventricle by opening and closing in a precisely coordinated manner. Functional mitral regurgitation, a back flow of blood from the ventricle into the atrium, is a clinical condition that can occur in response to myocardial infarction (Glower et al. 2014). In this situation, even though the mitral valve leaflets themselves are healthy, the valve fails to close properly because of geometric changes induced by the infarct such as papillary muscle displacement or ventricular enlargement (Rausch et al. 2017). The gold standard treatment to restore mitral valve function is to surgically implant an annuloplasty ring that constricts the mitral valve annulus and ensures sufficient leaflet coaptation (Amini et al. 2012). However, studies have shown that regurgitation recurs in more than half of the patients within three to five years after mitral valve repair (Flameng et al. 2003). A better understanding of the parameters that affect the mitral valve behavior would allow for a more efficient design by directly addressing the patient’s needs to improve the long-term repair outcome. The Living Heart Model (Baillargeon et al. 2014) allows us to simulate the integrated dynamic response of the heart in terms of its electrical, mechanical, and flow physics response. Because of its modular nature, we can easily modify and adjust the healthy baseline model to introduce personalized disease states and evaluate treatment opportunities by virtually implanting various types of devices.

**Fig. 9:**
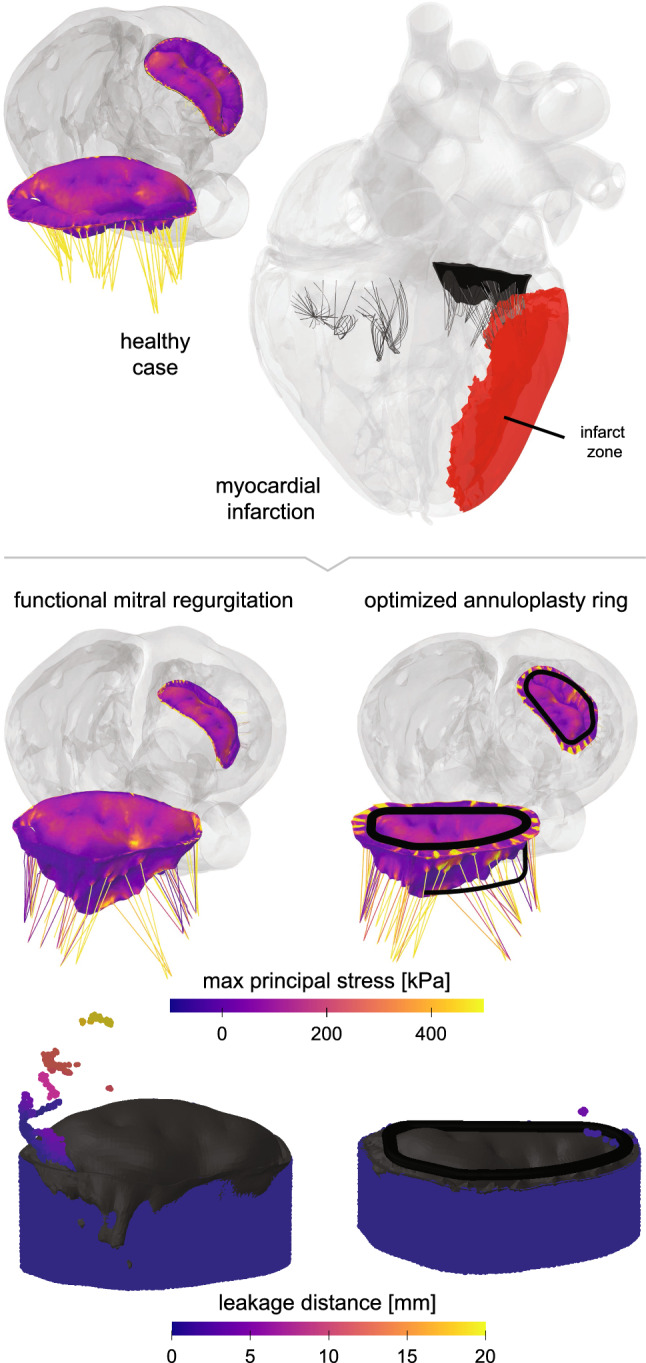
Cardiac mechanics—Annuloplasty. Maximum principal stresses in the mitral valve apparatus. On top left, the healthy baseline case with an appropriately closing valve is shown. On the top right, functional mitral regurgitation is created by simulating an infarct in the lateral left ventricular wall. When left untreated, this induced functional mitral regurgitation leads to the deformation and leakage shown in the mid and bottom left plots. The mid and bottom right plots showcase how an optimized annuloplasty ring can force the valve leaflets to close appropriately and counteract any serious regurgitation

### Simulation

We induce functional mitral regurgitation through myocardial infarction in the Living Heart Model by modifying the active material properties in the infarcted region while leaving the passive material properties unchanged. By varying the size of the modified region, we can induce degrees levels of myocardial infarction causing different degrees of mitral regurgitation. Figure 9 shows the infarcted region of our simulation affecting the lateral left ventricular wall. This type of myocardial infarction results in an asymmetric behavior of the mitral valve because it selectively displaces a group of chordae, while others remain in their physiological position. In response, as Fig. 9 suggests, the mitral valve no longer closes properly. To virtually reduce the degree of regurgitation and repair the leaking valve, we implant an annuloplasty ring. We select a ring with a sub-valvular component (Baillargeon et al. 2015) to restore mitral valve function. We approximate the ring as rigid to focus on the deformation of the valve. We insert the ring into the 70% diastolic heart and then suture it onto to the mitral annulus using axial connecter elements (Rausch et al. 2017). Following the implantation, we optimize the degree of leakage using Isight to adjust three geometrical parameters of the ring, the height and length of the subvalvular element and the radius of curved segment (Isight 2020). We define the degree of leakage as the number of particles passing through a threshold plane parallel to the annulus using smoothed particle hydrodynamics simulations. Since this analysis is a post-processing step, the particles from the smoothed particle hydrodynamics simulations do not affect the behavior of the mitral valve and purely serve to quantify the degree of the leakage (Abaqus 2020). The number of passing particles defines the objective function, which we minimize throughout the optimization process.

### Discussion

Our device design study indicates that, among the three design variables, the strongest effect is from changing the length, which is likely due to different chordae being engaged with various lengths. This highlights the importance of identifying the appropriate chordae that need to be included or excluded in the engagement with the ring to minimize mitral regurgitation. Based on the myocardial infarction model and the ring design in this study, the engagement of marginal chordae that attach to the P3 scallop seems to affect the A3-P3 coaptation segment while the basal posterior chordae are engaged to improve the A2-P2 coaptation. After the initial optimization study, we selected the best design with the smallest objective function as the new starting point for further design optimization using the downhill simplex method (Isight 2020). This secondary optimization constrains the three design variables within $\pm 20\%$ of their initial best design values. Figure 9 summarizes the results of the optimization. It suggests that the degree of mitral regurgitation was significantly reduced after virtually implanting the device with the best design. This study demonstrates that personalized finite element simulations–embedded in a systematic optimization algorithm–provide a powerful technology to better understand valvular functioning in healthy hearts and introduce mitral regurgitation to optimize device dimensions and repair efficiency. Predictive personalized simulations of surgical intervention, like the one we have shown here, have the potential to optimize surgical procedures, improve device design, and guide treatment planning on an individual personalized basis.

## Fluid–structure interaction

**Fig. 10:**
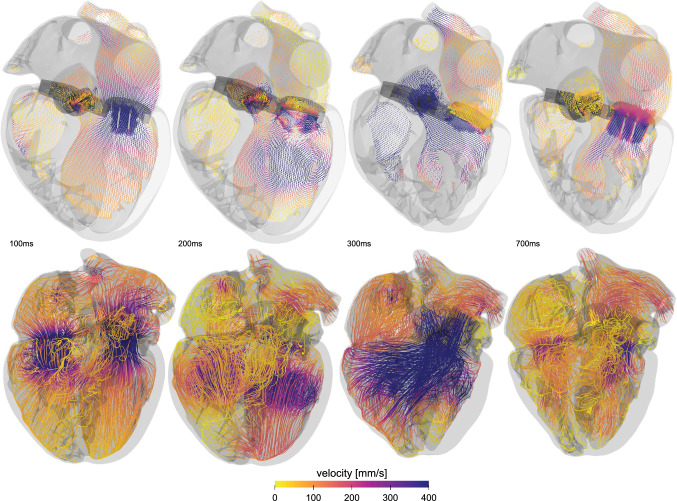
Fluid–structure interaction in the heart. Velocity profiles illustrate the blood flow at different time points throughout the cardiac cycle. Streamlines highlight locations of high and low velocity and provide insight into regions of high thrombogenic risk. The velocities are highest during systole, when they locally exceed 400 mm/s

### Motivation

In understanding physiological and pathological conditions of the human heart, high fidelity multi-physics models of hemodynamic phenomena are of critical importance. Depending on the type of application, it is often sufficient to represent the pump function of the heart through one-dimensional fluid network models that accurately capture the dynamic pressure-volume changes in the intra- and extra-cardiac circulation (Baillargeon et al. 2014). However, when shear stresses on the cardiac wall, the leaflets, the annuli, or newly implanted devices play a relevant role (Cherubini et al. 2015), it is crucial to correctly capture hemodynamic phenomena and their impact on the relevant structures (Gizzi et al. 2011). Multiphysics simulations of the heart that include both solid and fluid (Nordsletten et al. 2011) often build on technologies developed for other engineering applications including aerospace, energy, or defense. From a purely computational fluid dynamics perspective, the major challenges of cardiac simulations consist of the discretization of space and time, the characterization of initial and boundary conditions, and the large deformations associated with the contraction and relaxation of the heart. Fluid structure interaction introduces additional challenges associated with mapping between non-conforming computational grids, accurate surface modeling, and computational efficiency. The integration of a fully Navier-Stokes based commercial computational fluid dynamics software, FlowVision, with Abaqus/Explicit proposes several solutions for these challenges in cardiac simulations (Aksenov 2017). This approach uses a sub-grid geometry resolution method (Aksenov et al. 1998) from the native computational fluid dynamics simulation and constructs a new mesh at every discrete time point throughout the cardiac cycle. The time-dependent boundary conditions follow the inlet and outlet conditions of the original Living Heart Model (Baillargeon et al. 2014). For the fluid structure interaction calculations, an explicit coupling scheme is combined with pressure exchange surface management. For simplicity, the initial integration between the computational fluid dynamics software and the heart model does not include the valves. While this simplification does not impact the practical applicability of the model for device design or surgical procedures, it presents several opportunities for future upgrades: First a fully three-dimensional computational fluid dynamics simulation coupled with the whole heart model that includes mechanically deformable valves (Pisano et al. 2020), which move as a result of hemodynamic forces and second a fully coupled fluid-structure interaction model in which the flow causes the motion of the valves (Meschini et al. 2020), which move and deform and influence the fluid flow. Including deformable valves in a fully two-directional, strongly coupled approach has been a major milestone that made the simulated fluid-structure interaction a readily usable tool for many scientific applications (Aksenov et al. (2020).

### Simulation

In the fluid structure interaction approach (Aksenov et al. 2006; Aksenov 2017; Sodhani et al. 2018) the solid deformable parts such as the heart valves are allowed to move and come closer together. A recent overview of blood flow modeling in the beating human heart (D’Souza et al. 2018) discusses different computational fluid dynamics strategies including Navier-Stokes, Lattice-Boltzman, and Smoothed Particle Hydrodynamics. Of these, Navier Stokes based approaches seem best suited for complex valve geometries with tiny gaps, complex mesh generation, and large deformations. To simulate the heart valve dynamics in the living human heart we divide the computational domain dynamically into two or more substructures to accurately model partial or full contact and closure. When the opening ratio, the ratio between the largest and the smallest opening section, is too large, the computational fluid dynamics algorithm encounters dimensionality problems. To accurately resolve the flow, the discretization needs to be extremely fine. To address this problem, the Navier Stokes based three-dimensional fluid algorithm uses a reduced order approach referred to as Gap Model. Originally developed for screw compressor applications (Ozturk et al. 2019), the Gap Model approach has been validated under different flow conditions. In our simulation, when two solid structures come close to one another and eventually exceed the proximity threshold, the Gap Model is activated automatically. This allows us to conduct fluid structure interaction studies in practically feasible time ranges. In Fig. 10, we combine a one-way fluid structure interaction to estimate myocardial movements and a two-way strongly coupled fluid-structure interaction to ensure high accuracy of the device simulations.

### Discussion

To understand the forces that act on heart valve leaflets, it is critical to not only study the fluid or the structure in complete isolation, but to understand how the blood flow modulates the shape of the leaflets and, in turn, how the motion of the leaflets modulates the flow conditions. A recent study that compared finite element analysis and fluid structure interaction found that, in agreement with in-vitro studies of artificial aortic heart valves, the fluid structure interaction approach predicts higher effective stresses in the leaflet belly and edge regions (Sodhani et al. 2018). These higher stresses are a result of the hammer effect, which naturally cannot be captured by decoupled finite element analyses. A similar comparative study revealed that only the fluid structure interaction approach was able to accurately capture the asymmetric opening and closing of the valve (Luraghi et al. 2017). A recent study investigated the performance and complications of a fluid structure interaction approach in combination with the Living Heart Model to study transcatheter aortic valve replacement (Ghosh et al. 2020). To validate the model, the study used personalized post transcatheter aortic valve replacement echo Doppler measurements. This suggests that we can use computer tomography images to reconstruct personalized models and adopt a fluid structure interaction approach to study the effect of valve deployment and positioning on stent anchorage in both self-expanding and balloon inflated transcatheter aortic valves. Another important problem that requires high resolution fluid simulations is thrombogenicity. From a computational fluid dynamics perspective, characterizing thrombogenic risk requires an accurate prediction of wall shear stresses and fluid shear stresses. This is often achieved by using the exact finite element surface in a finite volume representation to augment the spatial resolution (Aksenov et al. 1998). Alternatively, several studies have proposed to use smoothed particle hydrodynamics (Mao et al. 2016, 2017; Caballero et al. 2017). These studies suggest that wall shear stresses and velocity gradients are not satisfactorily resolved, which makes this approach unsuitable for thrombogenic risk assessment. Other factors, including the inability to model incompressibility and limitations regarding flow and pressure waveforms, are additional common points of criticism. A recent thrombogenic analysis of a 29 mm $\hbox {CoreValve}^\text {TM}$ (Medtronic, Santa Rosa), deployed at annular versus supra-annular locations, of a personalized heart geometry found notable differences in the hemodynamics in the ascending aorta and the coronary arteries (Kandail et al. 2018). The study identified regions of high wall shear stresses at locations of para-valvular flow. In conclusion, the rapid developments in cardiac simulations throughout the past decade now enable us to perform practically suitable multiphysics simulations. While most the studies have been validated in an in-vitro setting, an urgent need remains to validation the simulations in vivo, for example, by using four-dimensional magnetic resonance imaging. The non-invasive nature and high resolution make four-dimensional magnetic resonance imaging ideally suited to personalize the fluid-structure-interaction simulations with a view towards personalized thrombogenic risk assessment and personalized device selection.

## Clinical perspective—Virtual imaging trials

**Fig. 11:**
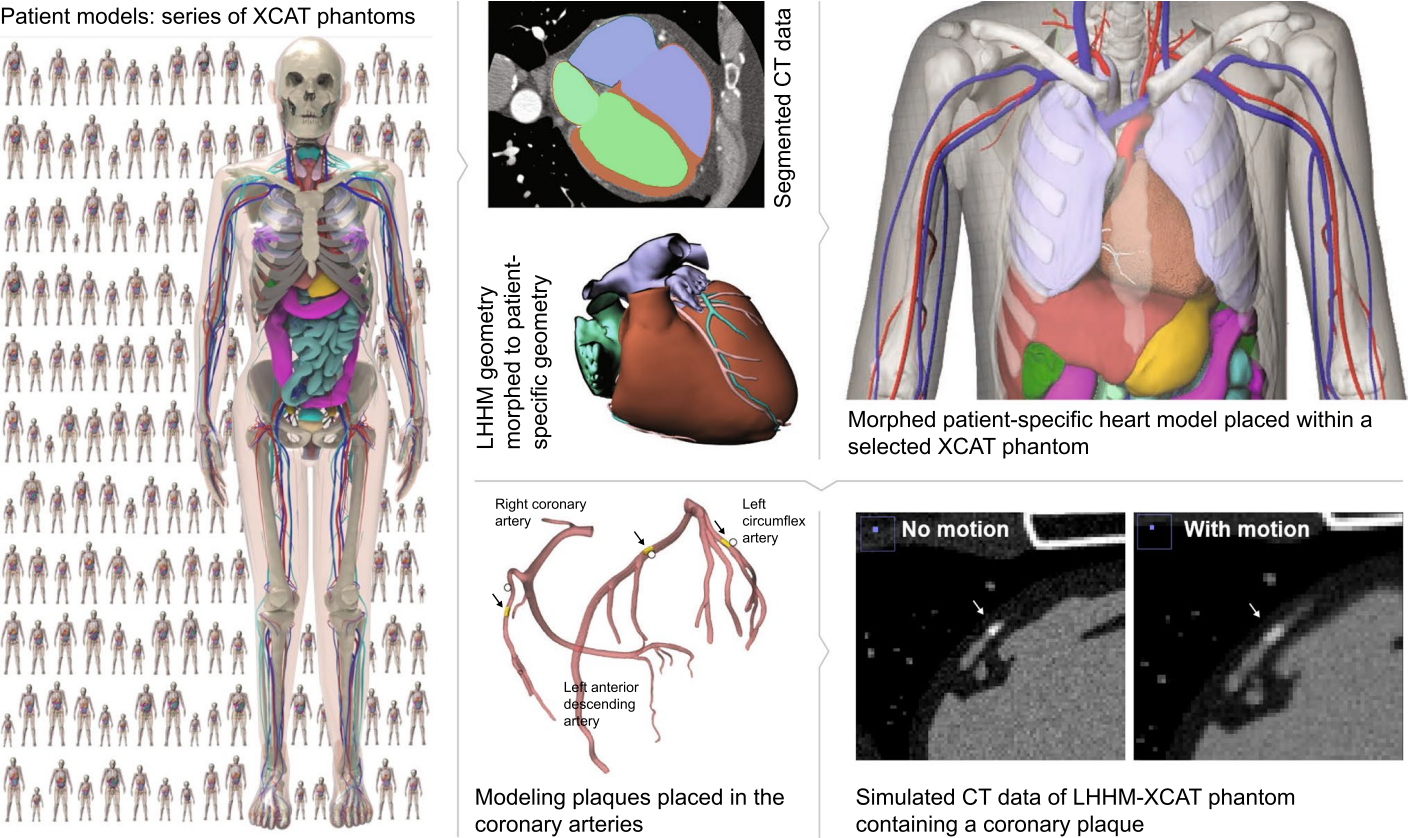
Clinical perspective—Virtual imaging trials. Population of whole-body phantoms, left. Anatomically variable human heart models are created by morphing the template Living Heart Model geometry to fit segmented patient-specific computer tomography data, top right. The new personalized models are placed within selected phantoms. To simulate coronary artery disease, plaques of any given size or material definition can be placed within the coronary vessels. The phantoms models can then be imaged with our computer tomography simulation framework to produce imaging data under various devices or parameters, bottom right. Simulated computer tomography data with and without cardiac motion during mid-diastole illustrate the plaque

### Motivation

Coronary artery disease is the number one cause of death for both men and women in the United States every year (Go et al. 2013). Medical imaging is integral to the diagnosis and management of coronary artery disease fueling the development of new technologies and applications. As new techniques emerge, a major challenge is how to devise their most effective use to optimally benefit the patient while minimizing any potential harm. Clinical trials are the best avenue to evaluate imaging devices and methods, but the ever-expanding number of technologies and parameters make a trial for every application or protocol unfeasible, both pragmatically and financially. Simulation-based virtual imaging trials can address this growing critical need. Virtual imaging trials involve the use of computational tools to perform experiments entirely on the computer. Such techniques are being widely investigated for breast imaging research (Das et al. 2009; Gong et al. 2006; Young et al. 2013). In a virtual imaging trial, realistic patient models or phantoms are imaged with validated simulation algorithms, modeling the imaging process and device, to emulate imaging examinations under different devices and parameters. From simulated images, we can investigate how differing patient attributes and imaging conditions impact dose, image quality, and the depiction of pre-defined known conditions. As such, virtual imaging trials can perform preclinical optimization on a growing number of new technologies with diverse attributes, helping to identify the most promising systems or system parameters for further clinical validation. To be truly effective, virtual imaging trials require realistic computational models of patients to serve as the known truth as well as accurate imaging simulation tools with which to image the virtual patients. Such tools are currently lacking in cardiac imaging research as existing phantoms do not realistically simulate variations in cardiac anatomy or function indicative of the population at large. In addition, current simulation tools are limited in their ability to model modern imaging devices. Our goal is to develop the essential tools to conduct virtual imaging trials in cardiac imaging research. Initially, we focus on computed tomography as it has great potential to provide high spatial and temporal resolution for the optimized evaluation of coronary artery disease.

### Simulation

To create realistic, anatomically variable computational phantoms to conduct virtual imaging trials in cardiac imaging, we combine the Living Heart Model (Baillargeon et al. 2014) with a four-dimensional extended cardiac-torso phantom library. This library showcased in Fig. 11 left, consists of a population of 150 computational whole-body models that represent both sexes with different ages, heights, and weights (Segars et al. 2013, 2015). To create anatomically variable heart models to use with the extended cardiac-torso, we utilize warping methods to non-rigidly deform the original heart model to fit diverse cardiac geometries based on four dimensional computed tomography patient data (Veress et al. 2019), see Fig. 11 middle. We incorporate the new heart models into different extended cardiac-torso phantom anatomies using an established mapping pipeline (Segars et al. 2019). The result is a library of whole-body anatomies with realistic, anatomically variable cardiac models with an added physiological basis, provided within the original Living Heart Model framework, as described, in the previous sections, to simulate variations in cardiac function. To image the cardiac-torso virtual patients, we develop and validate an analytical computed tomography simulator (Segars et al. 2008) as well as a next generation simulator, called DukeSim (Abadi et al. 2019) that combines analytical and Monte Carlo techniques to more accurately model the geometry and physics of commercial computed tomography scanners. Both simulators can generate data from dynamic four-dimensional computational phantoms and enable studies in the context of motion. Figure 11, right, shows an example computed tomography simulation from such a cardiac-torso phantom (Segars et al. 2019). We added a plaque of 50% blockage within the right coronary artery of an adult male extended cardiac-torso model and imaged it with and without an average cardiac motion during mid-diastole. The motion of the heart, although reduced during mid-diastole, can still be seen to cause a reduction in the contrast of the plaque.

### Discussion

This study offers a major advance to enable realistic virtual imaging trials in cardiac imaging. Combining a prototype human heart model with extended cardiac-torso whole body models, we created a new series of realistic four dimensional computational phantoms. We have shown that different hearts can be integrated into different background anatomies to provide a diverse collection of phantoms capable of simulating a range of normal and abnormal cardiac anatomies and functions. To image the phantoms, we created an accurate computed tomography simulation package capable of simulating realistic cardiac computed tomography data from various manufacturers. This computational platform provides the essential tool to quantitatively optimize clinical cardiac technologies in terms of image quality and radiation dose for more precise and personalized imaging of the heart. As illustrated in Fig. 11, this platform can be used to investigate imaging methods in comparison to a known ground truth, for example, the precise location and size of a plaque. With a view towards precision medicine, we can easily adjust the anatomical, physiological, and imaging parameters of our model and quantify their effects by comparing the simulated images to the known truth defined within the phantom or virtual patient. Such studies are not feasible using real human subjects because of ethical concerns over radiation exposure and the lack of a ground truth. Initially, this study focusses on computed tomography due to its potential importance in coronary artery disease evaluation. Long term, our goal is to extend our platform towards introducing precision medicine into other cardiac conditions, imaging modalities, and simulation technologies.

## Regulatory perspective—Medical device innovation

### Motivation

Digital twins, virtual patients based on computational modeling and simulation, have advanced as an important technology to improve efficiency of clinical trials for new device designs (Madni et al. 2019; Corral-Acero et al. 2020). In the context of cardiac device design, efforts are underway to explore the potential of human heart simulators as a digital evidence for new cardiovascular device approvals. The objectives of these in silico clinical trials are to reduce animal testing and to minimize the number of required patients while still ensuring safety and efficacy of the novel device (Sturla et al. 2017). A major advantage of this new digital process is that it is more efficient and less expensive than current excessive clinical trials whose delays and costs often impede patient access to novel treatments. At the same time, it is critical that the digital process is designed without loss of rigor or confidence in the safety and efficacy of the new device. Regulatory agencies are increasingly recognizing the public health benefits of modeling and simulation and the potential for in silico clinical trials to safely advance medical products more efficiently, from preclinical studies through clinical trials to market: Modeling and simulation can help to inform clinical trial designs, support evidence of effectiveness, identify the most relevant patient groups to study, and assess product safety (Alber et al. 2019). In some cases, in silico clinical trials have already shown to produce similar results as human clinical trials. For decades, in silico trails have been successfully used in regulated industries such as aerospace and automotive. In biomedicine, we are now recognizing the power of virtual patients to develop therapies for the heart, the vasculature, or the brain by eliminating traditional cost and time bottlenecks (Madni et al. 2019).

**Fig. 12:**
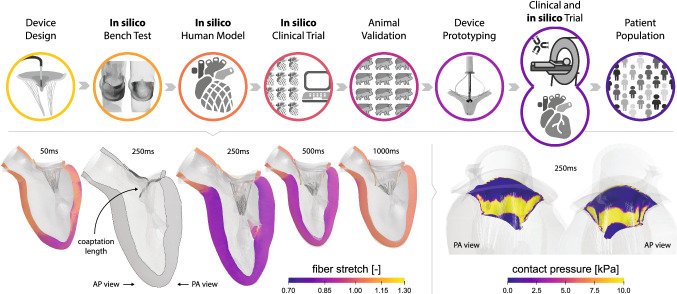
Transformative impact—Medical device innovation. Design of the ENRICHMENT trial, and fiber stretch and contact pressure profiles of baseline model at different time points throughout the cardiac cycle. The trial personalizes the baseline model by accounting for each patient’s individual degree of ventricular dilation, ejection fraction, annular dilation, and papillary muscle displacement. The trial uses early in silico testing and later animal validation and device prototyping

The ENRICHMENT trial, a new in silico clinical trial for cardiac device design, combines digital evidence from simulations with physical evidence from real patients and assesses model credibility in accordance with engineering standards. Figure 12 illustrates the workflow of the trial based on early in silico testing in realistic human environments and later animal validation and device prototyping. The trial focuses on functional mitral regurgitation and its treatment using edge-to-edge repair (Fucci et al. 1995). The $\hbox {MitraClip}^\text {TM}$ (Abbott, Santa Clara) is currently the sole percutaneous device that is commercially approved to treat functional mitral regurgitation and more than 80,000 patients have undergone repair with this device in the past decade (Abbott 2019). To reduce regurgitation, the device approximates the anterior and posterior mitral valve leaflets to create a double-barrel mitral orifice, a process that is relatively straightforward to simulate in a finite element setting (Zhang et al. 2019). The process to assess credibility of these simulations can be classified into three categories: First, the approach should show a general agreement between the expected behavior and the predicted metrics. Second, for appropriately calibrated model parameters, the approach should produce agreement between model and experiment. Third, for general validation, the model should agree with the experiment for no specific context of use. Ultimately, this implies that the model should demonstrate predictive behavior for a specific context of use (Gray and Pathmanathan 2018).

### Simulation

The objective of the ENRICHMENT trial is to explore the merits of designing and virtually testing a medical device in its end-use environment before building and physically testing prototypes. At the same time, it studies the possibility of utilizing data generated in virtual patients as credible evidence in a regulatory device review. The trial uses physics-based computational models and simulations of the device, implanted within a cohort of beating heart models, constructed to represent the disease state and other inclusion criteria in a virtual patient population. To create and validate these virtual patient models, the trial uses clinical information from real patients. Statistical variations from the validation set will be used to represent the target patient population. Initially, for proof of concept, the ENRICHMENT trial focuses on the condition of functional mitral regurgitation and on a patient population that shows acute success: Patients with severe mitral regurgitation, small ventricles, good ejection fraction, and a history with optimized medical management of heart failure. To accurately simulate the clinical endpoints of these functional mitral regurgitation patients, their computational models include their annulus, their mitral valve, their papillary muscles, their chordae, and their left ventricle simulated through the complete cardiac cycle. The constitutive behavior, hemodynamic loading, initial and boundary conditions are similar to the baseline model in Sect. 4. Figure 12 shows an anterolateral slice of the heart with its leaflets throughout the cardiac cycle. The color code indicates the fiber stretch. The antero-lateral view highlights the coaptation length and the zoom in shows the corresponding contact pressure on the leaflets at 250ms, at peak systole. The simulated mitral valve has mid-diastolic and mid-systolic anterio-posterior diameters of 35.4mm and 28.6mm, intercomissural diameters of 40.3mm and 37.2mm, circumferences of 121mm and 110mm, and areas of $1132\hbox {mm}^2$ and $8562\hbox {mm}^2$. These dimensions are consistent with the clinically observed norms of healthy patients (Grewal et al. 2010; Lee et al. 2013; Sturla et al. 2017). The billowing and tenting heights of 1.6mm and 4.4mm and the coaptation length of 10mm at peak systole also agree with the healthy reported ranges (Mihaila et al. 2016; Sturla et al. 2017). The ENRICHMENT trial personalizes this healthy human heart model by accounting for each patient’s individual degree of ventricular dilation, ejection fraction, annular dilation, and papillary muscle displacement. To incorporate the backward flow of regurgitation, the personalized hemodynamic model accounts for the individual dynamics of the regurgitant orifice area and the shape of the mitral valve (Cohen and Gorlin 1972). Each personalized model undergoes a virtual edge-to-edge repair with the virtual device, under the guidance of a clinical advisory board to replicate the judgement used in a real clinical trial (Dabiri et al. 2019). For each virtual repair process, the ENRICHMENT trial evaluates the relevant clinical readouts including the coaptation height, the residual regurgitation, the spreading force on the clipping device, and the left atrial pressure. The in silico clinical trial also allows access to other metrics that are not immediately accessible in a physical clinical trial, but can provide valuable physiological insight to quantify the success of the repair process.

### Discussion

The general objective of this study is to raise awareness and capability for others to perform in silico clinical trials, agnostic to the software tools. In contrast to classical clinical trials that use simulation as a post-processing tool for data analysis, the ENRICHMENT trial uses silico testing early on in the workflow to put less patients at risk, save time and resources, and provide better designs with a faster turnaround time. The specific objective of the trial is to demonstrate how digital evidence, in the form of virtual patients, can be used to significantly reduce the time, cost, and risk with human clinical trial data collection. On a broader scale, this study demonstrates that a collaborative product lifecycle management platform can significantly improve the robustness, response time, and transparency of the medical device review process. It enables regulators with full digital access to all relevant information and people required to make science-based, informed regulatory decisions. Several limitations of the current study design point towards potential future improvements. An important missing link is the longitudinal assessment of chronic disease management. From an analysis point of view, this involves not only simulate and analyze the acute changes in the stretch and stress profiles in response to the intervention (Zhang et al. 2019), but to predict and understand the chronic changes in form and function (Rausch et al. 2017). Another future direction is to integrate simulation results, animal models, and clinical data using physics-based machine learning towards a more holistic understanding of disease progression and the long-term effects of intervention. Bringing together researchers, medical doctors, surgeons, device design manufacturers, and regulatory agencies is a first step in this direction with the common goal to safely improve device design, accelerate device approval, and, ultimately, improving human health.

## Conclusion

With the rapid developments in machine learning, data-driven modeling, and physics-based simulation, we can confidently assume that, within the next decade, we will be able to simulate each person’s individual heart. Within this perspectives article, we show what is already possible today, what will be possible in the near future, and what will probably never be possible. We have identified the missing pieces to create fully personalized, high-resolution whole heart models that encode a fully personalized medical history. While conceptually possible, this process is technically cumbersome, computationally expensive, and labor intensive. Instead, we propose to create personalized human heart models out of population-based libraries with geometric, biological, physical, and clinical information by morphing between a finite number of fully reconstructed four-dimensional human heart models using machine learning. We propose to analyze these personalized human heart models by combining physics-based multiscale modeling and machine learning. This allows us to learn the underlying physics, infer the model parameters, analyze parameter sensitivities, and quantify model uncertainties. We have outlined the challenges and opportunities for precision medicine in human heart modeling, not only as a purely academic exercise, but also as a translational path towards clinical decision making in full alignment with the regulatory agencies. Our examples highlight the potential for personalized human heart simulations in medical device design, clinical decision making, and personalizing treatment planning.

## References

[CR1] Abadi E, Harrawood B, Sharma S, Kapadia A, Segars WP, Samei E (2019). Dukesim: a realistic, rapid, and scanner-specific simulation framework in computed tomography. IEEE Transac Med Imaging.

[CR2] Abaqus Analysis User’s Guide. Dassault Systèmes Simulia Corp., 2020

[CR3] Abbott Press Resease. Abbott receives FDA approval for expanded indication for MitraClip^TM^ device. https://abbott.mediaroom.com/2019-03-14-Abbott- Receives-FDA-Approval-for-Expanded-Indication-for -MitraClip-TM-Device. published: Mar 14, 2019

[CR4] Aksenov A, Dyadkin A, Pokhilko V (1998). Impact of annular and supra-annular $$\text{CoreValve}^\text{ TM }$$ deployment locations on aortic and coronary artery hemodynamics. ASME 1998 Press Vess Piping Conf Num.

[CR5] Aksenov A, Iliine K, Luniewski T, McArthy T, Popielas F, Ramkumar R (2006) Oil leakage through a valve stem seal. Proceedings Abaqus User Conference

[CR6] Aksenov A (2017). Flowvision: industrial computational fluid dynamics. Comp Res Model.

[CR7] Aksenov A, Zhluktov S, Zietak W, Cotton R, Vučinić D (2020) Human heart blood flow numerical modelling and simulations. Lecture Notes in Mechanical Engineering, pp 237–263 Springer, Singapore

[CR8] Alber M, Buganza Tepole A, Cannon WR, De S, Dura-Bernal S, Garikipati K, Karniadakis G, Lytton WW, Perdikaris P, Petzold L, Kuhl E (2019). Integrating machine learning and multiscale modeling: perspectives, challenges, and opportunities in the biological, biomedical, and behavioral sciences. npj Digital Med.

[CR9] Aliev RR, Panfilov AV (1996). A simple two-variable model of cardiac excitation. Chaos Solitons Fract.

[CR10] American Heart Association (2020) Heart Disease and Stroke Statistics–2020 Update. American Heart Association, Dallas, Texas

[CR11] Amini R, Eckert CE, Koomalsingh K, McGarvey J, Minakawa M, Gorman JH, Gorman RC, Sacks MS (2012). On the in vivo deformation of the mitral valve anterior leaflet: effects of annular geometry and referential configuration. Ann Biomed Eng.

[CR12] Ambrosi D, Arioli G, Nobile F, Quarteroni A (2011). Electromechanical coupling in cardiac dynamics: the active strain approach. SIAM J Appl Math.

[CR13] Ambrosi D, Ben Amar M, Cyron CJ, DeSimone A, Goriely A, Humphrey JD, Kuhl E (2019). Growth and remodelling of living tissues: perspectives, challenges and opportunities. J Royal Soc Interf.

[CR14] Aguado-Sierra J, Krishnamurthy A, Villongco C, Chuang J, Howard E, Gonzales MJ, Omens J, Krummen DE, Narayan S, Kerckhoffs RC, McCulloch AD (2011). Patient-specific modeling of dyssynchronous heart failure: a case study. Prog Biophys Mole Biol.

[CR15] Baillargeon B, Rebelo N, Fox DD, Taylor RL, Kuhl E (2014). The living heart project: a robust and integrative simulator for human heart function. European J Mech A/Solids.

[CR16] Baillargeon B, Costa I, Leach JR, Lee LC, Genet M, Toutain A, Wenk JF, Rausch MK, Rebelo N, Acevedo-Bolton G, Kuhl E, Navia JL, Guccione JM (2015). Human cardiac function simulator for the optimal design of a novel annuloplasty ring with a sub-valvular element for correction of ischemic mitral regurgitation. Cardiovas Eng Technol.

[CR17] Bayer JD, Blake RC, Plank G, Trayanova NA (2012). A novel rule-based algorithm for assigning myocardial fiber orientation to computational heart models. Ann Biomed Eng.

[CR18] Bom MJ, Levin E, Driessen RS, Danad I, Van Kuijk CC, van Rossum AC, Narula J, Min JK, Leipsic JA, Pereira JPB, Taylor CA (2019). Predictive value of targeted proteomics for coronary plaque morphology in patients with suspected coronary artery disease. EBioMedicine.

[CR19] Bordas R, Gillow K, Lou Q, Efimov IR, Gavaghan D, Kohl P, Grau V, Rodriguez B (2011). Rabbit-specific ventricular model of cardiac electrophysiological function including specialized conduction system. Prog Biophys Mole Biol.

[CR20] Caballero A, Mao W, Liang L, Oshinski J, Primiano C, McKay R, Kodali S, Sun W (2017). Modeling left ventricular blood flow using smoothed particle hydrodynamics. Cardiovas Eng Technol.

[CR21] Çetingül HE, Plank G, Trayanova N, Vidal R (2011). Estimation of local orientations in fibrous structures with applications to the Purkinje system. IEEE Transac Biomed Eng.

[CR22] Chabiniok R, Wang VY, Hadjicharalambous M, Asner L, Lee J, Sermesant M, Kuhl E, Young AA, Moireau P, Nash MP, Chapelle D, Nordsletten DA (2016). Multiphysics and multiscale modelling, data-model fusion and integration of organ physiology in the clinic: ventricular cardiac mechanics. Interf Focus.

[CR23] Cherubini C, Filippi S, Gizzi A, Nestola MGC (2015). On the wall shear stress gradient in fluid dynamics. Commun Comput Phys.

[CR24] Cherubini C, Filippi S, Gizzi A, Ruiz-Baier R (2017). A note on stress-driven anisotropic diffusion and its role in active deformable media. J Theoret Biol.

[CR25] Cherry EM, Fenton FH (2012). Contribution of the Purkinje network to wave propagation in the canine ventricle: insights from a combined electrophysiological-anatomical model. Nonlinear Dyn.

[CR26] Cohen MV, Gorlin R (1972). Modified orifice equation for the calculation of mitral valve area. Am Heart J.

[CR27] Colatsky T, Fermini B, Gintant G, Pierson JB, Sager P, Sekino Y, Strauss DG, Stockbridge N (2016). The comprehensive in vitro proarrhythmia assay (CiPA) initiative – update on progress. J Pharmacol Toxicol Methods.

[CR28] Corrado C, Niederer SA (2016). A two-variable model robust to pacemaker behaviour for the dynamics of the cardiac action potential. Math Biosci.

[CR29] Corral-Acero J, Margara F, Marciniak M, Rodero C, Loncaric F, Feng Y, Gilbert A, Fernandes JF, Bukhari HA, Wajdan A, Martinez M Villegas, Santos M Sousa, Shamohammdi M, Luo H, Westphal P, Leeson P, DiAchille P, Gurev V, Mayr M, Geris L, Pathmanathan P, Morrison TM, Cornelussen R, Prinzen F, Delhaas T, Doltra A, Sitges M, Vigmond EJ, Zacur E, Grau V, Rodriguez B, Remme EW, Niederer S, Mortier P, McLeod K, Potse M, Pueyo E, Bueno-Orovio A, Lamata P (2020) The ‘Digital Twin’ to enable the vision of precision cardiology. European Heart Journal, ehaa159 10.1093/eurheartj/ehaa159 PMC7774470 32128588

[CR30] Crumb WJ, Vicente J, Johannesen L, Strauss DG (2016). An evaluation of 30 clinical drugs against the comprehensive in vitro proarrhythmia assay (CiPA) proposed ion channel panel. J Pharmacol Toxicol Methods.

[CR31] Cyron CJ, Humphrey JD (2017). Growth and remodeling of load-bearing biological soft tissues. Meccanica.

[CR32] Dabiri Y, Yao J, Sack KL, Kassab GS, Guccione JM (2019). Tricuspid valve regurgitation decreases after MitraClip implantation: fluid structure interaction simulation. Mech Res Commun.

[CR33] Dal H, Göktepe S, Kaliske M, Kuhl E (2012). A fully implicit finite element method for bidomain models of cardiac electrophysiology. Comput Methods Biomech Biomed Eng.

[CR34] Dang NC, Topkara VK, Mercando M, Kay J, Kruger KH, Aboodi MS, Oz MC, Naka Y (2006). Right heart failure after left ventricular assist device implantation in patients with chronic congestive heart failure. J Heart Lung Transplant.

[CR35] Das M, Gifford HC, O’Connor JM, Glick SJ (2009). Evaluation of a variable dose acquisition technique for microcalcification and mass detection in digital breast tomosynthesis. Med Phys.

[CR36] Dessertenne F (1966). La tachycardie ventriculaire a deux foyers opposes variables. Arch Mal Coeur Vaiss.

[CR37] D’Souza K, Butz B, Bianchi M, Ghosh R, Zietak W, Bluestein D (2018) Blood flow modeling in a beating human heart with applications in medical device design and patient care. Conference on Advancing Analysis and Simulation in Engineering (NAFEMS CAAS)

[CR38] Dubin D (1996) Rapid Interpret of EKG’s. Cover Publishing Company

[CR39] Eriksson TSE, Prassl AJ, Plank G, Holzapfel GA (2013). Modeling the disperson in electromechanically coupled myocardium. Int J Num Methods Biom Eng.

[CR40] Fenton FH, Cherry EM (2008). Models of cardiac cell. Scholarpedia.

[CR41] FitzHugh R (1961). Impulses and physiological states in theoretical models of nerve membrane. Biophys J.

[CR42] Flameng W, Herijgers P, Bogaerts K (2003). Recurrence of mitral valve regurgitation after mitral valve repair in degenerative valve disease. Circulation.

[CR43] Franzen O, van der Heyden J, Baldus S, Schlüter M, Schillinger W, Butter C, Hoffmann R, Corti R, Pedrazzini G, Swaans MJ, Neuss M, Rudolph V, Sürder D, Grünenfelder J, Eulenburg C, Reichenspurner H, Meinertz T, Auricchio A (2011). MitraClip® therapy in patients with end-stage systolic heart failure. European J Heart Fail.

[CR44] Fucci C, Sandrelli L, Pardini A, Torracca L, Ferrari M, Alfieri O (1995). Improved results with mitral valve repair using new surgical techniques. European J Cardio Thoracic Surg.

[CR45] Gee MW, Förster C, Wall WA (2010). A computational strategy for prestressing patient-specific biomechanical problems under finite deformation. Int J Num Methods Biomed Eng.

[CR46] Genet M, Lee LC, Nguyen R, Haraldsson H, Acevedo-Bolton G, Zhang Z, Ge L, Ordovas K, Kozerke S, Guccione JM (2014). Distribution of normal human left ventricular myofiber stress at end diastole and end systole: a target for in silico design of heart failure treatments. J Appl Physiol.

[CR47] Genet M, Rausch MK, Lee LC, Choy S, Zhao X, Kassab GS, Kozerke S, Guccione JM, Kuhl E (2015). Heterogeneous growth-induced prestrain in the heart. J Biomech.

[CR48] Genet M, Lee LC, Baillargeon B, Guccione JM, Kuhl E (2016). Modeling pathologies of diastolic and systolic heart failure. Ann Biomed Eng.

[CR49] Ghosh RP, Marom G, Bianchi M, D’Souza K, Zietak W, Bluestein D (2020). Numerical evaluation of transcatheter aortic valve performance during heart beating and its post-deployment fluid-structure interaction analysis. Biomech Model Mechanobiol.

[CR50] Gizzi A, Bernaschi M, Bini D, Cherubini C, Filippi S, Melchionna S, Succi S (2011). Three-band decomposition analysis of wall shear stress in pulsatile flows. Phys Rev E.

[CR51] Glower DD, Kar S, Trento A, Lim DS, Bajwa T, Quesada R, Whitlow PL, Rinaldi MJ, Grayburn P, Mack MJ, Mauri L, McCarthy PM, Feldman T (2014). Percutaneous mitral valve repair for mitral regurgitation in high-risk patients: results of the EVEREST II study. J Am Coll Cardiol.

[CR52] Go AS, Mozaffarian D, Roger VL, Benjamin EJ, Berry JD, Blaha MJ, Dai S, Ford ES, Fox CS, Franco S (2013). AHA statistical update. Circulation.

[CR53] Göktepe S, Abilez OJ, Kuhl E (2010). A generic approach towards finite growth with examples of athlete’s heart, cardiac dilation, and cardiac wall thickening. J Mech Phys Solids.

[CR54] Göktepe S, Abilez OJ, Parker KK, Kuhl E (2010). A multiscale model for eccentric and concentric cardiac growth through sarcomerogenesis. J Theor Biol.

[CR55] Göktepe S, Kuhl E (2010). Electromechanics of the heart: a unified approach to the strongly coupled excitation-contraction problem. Comput Mech.

[CR56] Göktepe S, Menzel A, Kuhl E (2014). The generalized Hill model: a kinematic approach towards active muscle contraction. J Mech Phys Solids.

[CR57] Gong X, Glick SJ, Liu B, Vedula AA, Thacker S (2006). A computer simulation study comparing lesion detection accuracy with digital mammography, breast tomosynthesis, and cone-beam ct breast imaging. Med Phys.

[CR58] Gray RA, Pathmanathan P (2018). Patient-specific cardiovascular computational modeling: diversity of personalization and challenges. J Cardiovas Transl Res.

[CR59] Grewal J, Suri R, Mankad S, Tanaka A, Mahoney DW, Schaff HV, Fletcher A, Sarano ME (2010). Mitral annular dynamics in myxomatous valve disease: new insights with real-time 3-dimensional echocardiography. Circulation.

[CR60] Grossman W (1980). Cardiac hypertrophy: useful adaptation or pathologic process?. Am J Med.

[CR61] Guccione JM, McCulloch AD, Waldman LK (1991). Passive material properties of intact ventricular myocardium determined from a cylindrical model. J Biomech Eng.

[CR62] Guccione JM, Costa KD, McCulloch AD (1995). Finite element stress analysis of left ventricular mechanics in the beating dog heart. J Biomech.

[CR63] Holzapfel GA, Ogden RW (2009). Constitutive modelling of passive myocardium: a structurally based framework for material characterization. Philos Transac Royal Soc A Math Phys Eng Sci.

[CR64] Hunter PJ, McCulloch AD, Ter Keurs HEDJ (1998). Modelling the mechanical properties of cardiac muscle. Prog Biophys Mole Biol.

[CR65] Hurtado DE, Castro S, Gizzi A (2016). Computational modeling of non-linear diffusion in cardiac electrophysiology: a novel porous-medium approach. Comput Methods Appl Mech Eng.

[CR66] Hurtado DE, Rojas G (2018). Non-conforming finite-element formulation for cardiac electrophysiology: an effective approach to reduce the computation time of heart simulations without compromising accuracy. Comput Mech.

[CR67] Ijiri T, Ashihara T, Yamaguchi T, Takayama K, Igarashi T, Shimada T, Namba T, Haraguchi R, Nakazawa K (2008). A procedural method for modeling the purkinje fibers of the heart. J Physiol Sci.

[CR68] Isight Documentation. Dassault Systèmes Simulia Corp., (2020)

[CR69] Jilberto J, Hurtado DE (2018). Semi-implicit non-conforming finite-element schemes for cardiac electrophysiology: a framework for mesh-coarsening heart simulations. Frontn Physiol.

[CR70] Jimenez JH, Forbess J, Croft LR, Small L, He Z, Yoganathan AP (2006). Effects of annular size, transmitral pressure, and mitral flow rate on the edge-to-edge repair: an in vitro study. Ann Thoracic Surg.

[CR71] Kaboudian A, Cherry EM, Fenton FH (2019). Real-time interactive simulations of large-scale systems on personal computers and cell phones: toward patient-specific heart modeling and other applications. Sci Adv.

[CR72] Kaiser AD, Shad R, Hiesinger W, Marsden AL (2020) A design-based model of the aortic valve for fluid-structure interaction arXiv preprint arXiv:2010.02346 10.1007/s10237-021-01516-7 PMC10752438 34549354

[CR73] Kandail HS, Trivedi SD, Shaikh AC, Bajwa TK, O’Hair DP, Jahangir A, LaDisa JF (2018). Impact of annular and supra-annular $$\text{ CoreValve}^{{\rm TM}}$$ deployment locations on aortic and coronary artery hemodynamics. J Mech Behav Biomed Mat.

[CR74] Karma A (2013). Physics of cardiac arrhythmogenesis. Ann Rev Condens Mat Phys.

[CR75] Kassab GS (2009). A systems approach to tissue remodeling. J Biomech Eng.

[CR76] Kavarana MN, Pessin-Minsley MS, Urtecho J, Catanese KA, Flannery M, Oz MC, Naka Y (2002). Right ventricular dysfunction and organ failure in left ventricular assist device recipients: a continuing problem. Ann Thoracic Surg.

[CR77] Kerckhoffs RC, Neal ML, Gu Q, Bassingthwaighte JB, Omens JH, McCulloch AD (2007). Coupling of a 3d finite element model of cardiac ventricular mechanics to lumped systems models of the systemic and pulmonic circulation. Ann Biomed Eng.

[CR78] Kerckhoffs RC, Omens J, McCulloch AD (2012). A single strain-based growth law predicts concentric and eccentric cardiac growth during pressure and volume overload. Mech Res Commun.

[CR79] Klingensmith ME (2008). The Washington Manual of Surgery.

[CR80] Kotsakou M, Kioumis I, Lazaridis G, Pitsiou G, Lampaki S, Papaiwannou A, Karavergou A, Tsakiridis K, Katsikogiannis N, Karapantzos I, Karapantzou C, Baka S, Mpoukovinas I, Karavasilis V, Rapti A, Trakada G, Zissimopoulos A, Zarogoulidis K, Zarogoulidis P (2015). Pacemaker insertion. Ann Translat Med.

[CR81] Krishnamurthy A, Villongco CT, Chuang J, Frank LR, Nigam V, Belezzuoli E, Stark P, Krummen DE, Narayan S, Omens JH, McCulloch AD (2013). Patient-specific models of cardiac biomechanics. J Comput Phys.

[CR82] Landajuela M, Vergara C, Gerbi A, Dedè L, Formaggia L, Quarteroni A (2018). Numerical approximation of the electromechanical coupling in the left ventricle with inclusion of the Purkinje network. Int J Num Methods Biomed Eng.

[CR83] Land S, Gurev V, Arens S, Augustin CM, Baron L, Blake R, Bradley C, Castro S, Crozier A, Favino M, Fastl TE, Fritz T, Gao H, Gizzi A, Griffith BE, Hurtado DE, Krause R, Luo X, Nash MP, Pezzuto S, Plank G, Rossi S, Ruprecht D, Seemann G, Smith NP, Sundnes J, Rice JJ, Trayanova N, Wang D, Wang ZJ, Niederer SA (2015). Verification of cardiac mechanics software: benchmark problems and solutions for testing active and passive material behaviour Proceedings of the Royal Society A: Mathematical. Phys Eng.

[CR84] Lee APW, Hsiung MC, Salgo IS, Fang F, Xie JM, Zhang YC, Lin QS, Looi JL, Wan S, Wong RH, Underwood MJ (2013). Quantitative analysis of mitral valve morphology in mitral valve prolapse with real-time 3-dimensional echocardiography: importance of annular saddle shape in the pathogenesis of mitral regurgitation. Circulation.

[CR85] Lim E, Dokos S, Cloherty SL, Salamonsen RF, Mason DG, Reizes JA, Lovell NH (2010). Parameter-optimized model of cardiovascular-rotary blood pump interactions. IEEE Transac Biomed Eng.

[CR86] Lombaert H, Peyrat JM, Croisille P, Rapacchi S, Fanton L, Cheriet F, Clarysse P, Magnin I, Delingette H, Ayache N (2012). Human atlas of the cardiac fiber architecture: study on a healthy population. IEEE Transac Med Imaging.

[CR87] Luraghi G, Wu W, De Gaetano F, Rodriguez-Matas JF, Moggridge GD, Serrani M, Stasiak J, Costantino ML, Migliavacca F (2017). Evaluation of an aortic valve prosthesis: fluid-structure interaction or structural simulation?. J Biomech.

[CR88] Luther S, Fenton FH, Kornreich BG, Squires A, Bittihn P, Hornung D, Zabel M, Flanders J, Gladuli A, Campoy L, Cherry EM (2011). Low-energy control of electrical turbulence in the heart. Nature.

[CR89] Lyon A, Mincholé A, Bueno-Orovio A, Rodriguez B (2019). Improving the clinical understanding of hypertrophic cardiomyopathy by combining patient data, machine learning and computer simulations: a case study. Morphologie.

[CR90] Madni AM, Madni CC, Lucerno SD (2019). Leveraging digital twin technology in model-based systems enginereering. Systems.

[CR91] Maeder MT, Leet A, Ross A, Esmore D, Kaye DM (2009). Changes in right ventricular function during continuous-flow left ventricular assist device support. J Heart and Lung Transpl.

[CR92] Mao W, Caballero A, McKay R, Primiano C, Sun W (2017). Fully-coupled fluid-structure interaction simulation of the aortic and mitral valves in a realistic 3D left ventricle model. PLoS ONE.

[CR93] Margara F, Wang ZJ, Levrero-Florencio F, Santiago A, Vázquez M, Bueno-Orovio A, Rodriguez B (2020) In-silico human electro-mechanical ventricular modelling and simulation for drug-induced pro-arrhythmia and inotropic risk assessment. Progress in Biophysics and Molecular Biology 10.1016/j.pbiomolbio.2020.06.007 PMC7848595 32710902

[CR94] Mao W, Li K, Sun W (2016). Fluid-structure interaction study of transcatheter aortic valve dynamics using smoothed particle hydrodynamics. Cardiovas Eng Technol.

[CR95] Mei Y, Hurtado DE, Pant S, Aggarwal A (2018). On improving the numerical convergence of highly nonlinear elasticity problems. Comput Methods Appl Mech Eng.

[CR96] Meschini V, Viola F, Verzicco R (2020). Heart rate effects on the ventricular hemodynamics and mitral valve kinematics. Comput Fluids.

[CR97] Mihaila S, Muraru D, Miglioranza MH, Piasentini E, Aruta P, Cucchini U, Iliceto S, Vinereanu D, Badano LP (2016). Relationship between mitral annulus function and mitral regurgitation severity and left atrial remodelling in patients with primary mitral regurgitation. European Heart J Cardiovas Imaging.

[CR98] Mirams GR, Niederer SA, Clayton RH (2020). The fickle heart: uncertainty quantification in cardiac and cardiovascular modelling and simulation. Philos Transac Royal Soc A Math Phys Eng Sci.

[CR99] Mulpuru SK, Madhavan M, McLeod CJ, Cha Y, Friedman PA (2017). Cardiac pacemakers: function, troubleshooting, and management. J Am Coll Cardiol.

[CR100] Nagumo J, Arimoto S, Yoshizawa S (1962). Active pulse transmission line simulating nerve axon. Proc Inst Radio Eng.

[CR101] Narayan SM, Krummen DE, Shivkumar K, Clopton P, Rappel WJ, Miller J (2012). Treatment of atrial fibrillation by the ablation of localized sources: the conventional ablation for atrial fibrillation with or without focal impulse and rotor modulation: CONFIRM trial. J Am Coll Cardiol.

[CR102] Nash MP, Hunter PJ (2000). Computational mechanics of the heart. J Elast Phys Sci Sol.

[CR103] Navarrete EG, Liang P, Lan F, Sanchez-Freire V, Simmons C, Gong T, Sharma A, Burridge PW, Patlolla B, Lee AS, Wu H, Beygui RE, Wu SM, Robbins RC, Bers DM, Wu JC (2013). Screening drug-induced arrhythmia using human induced pluripotent stem cell-derived cardiomyocytes and low-impedance microelectrode arrays. Circulation.

[CR104] Niederer SA, Kerfoot E, Benson AP, Bernabeu MO, Bernus O, Bradley C, Cherry EM, Clayton R, Fenton FH, Garny A, Heidenreich E, Land S, Maleckar M, Pathmanathan P, Plank G, Rodríguez JF, Roy I, Sachse FB, Seemann G, Skavhaug O, Smith NP (2011). Verification of cardiac tissue electrophysiology simulators using an N-version benchmark Philosophical Transactions of the Royal Society A: Mathematical. Phys Eng Sci.

[CR105] Nielsen PM, Le Grice IJ, Smaill BH, Hunter PJ (1991). Mathematical model of geometry and fibrous structure of the heart. Am J Physiol Heart Circ Physiol.

[CR106] Nielsen SL, Timek TA, Lai DT, Daughters GT, Liang D, Hasenkam JM, Ingels NB, Miller DC (2001). Edge-to-edge mitral repair: tension on the approximating suture and leaflet deformation during acute ischemic mitral regurgitation in the ovine heart. Circulation.

[CR107] Niestrawska JA, Augustin CM, Plank G (2020). Computational modeling of cardiac growth and remodeling in pressure overloaded hearts - Linking microstructure to organ phenotype. Acta Biomat.

[CR108] Nordsletten DA, Niederer SA, Nash MP, Hunter PJ, Smith NP (2011). Coupling multi-physics models to cardiac mechanics. Prog Biophys Mole Biol.

[CR109] O’Hara T, Virág L, Varró A, Rudy Y (2011). Simulation of the undiseased human cardiac ventricular action potential: model formulation and experimental validation. PLoS Comput Biol.

[CR110] Opie LH, Commerford PJ, Gersh BJ, Pfeffer MA (2006). Controversies in ventricular remodelling. The Lancet.

[CR111] Ozturk U, Soganci S, Akimov V, Tutkun O, Aksenov A (2019). Validation of FlowVision CFD on ICCS2015 test case: application of Gap Model and SGGR for leakage flow prediction in a dry screw compressor. IOP Conf Series Mat Sci Eng.

[CR112] Pathmanathan P, Bernabeu MO, Bordas R, Cooper J, Garny A, Pitt-Francis JM, Whiteley JP, Gavaghan DJ (2010). A numerical guide to the solution of the bidomain equations of cardiac electrophysiology. Prog Biophys Mole Biol.

[CR113] Peirlinck M, De Beule M, Segers P, Rebelo N (2018). A modular inverse elastostatics approach to resolve the pressure-induced stress state for in vivo imaging based cardiovascular modeling. J Mech Behav Biomed Mat.

[CR114] Peirlinck M, Sahli Costabal F, Sack KL, Choy JS, Kassab GS, Guccione JM, De Beule M, Segers P, Kuhl E (2019). Using machine learning to characterize heart failure across the scales. Biomech Model Mechanobiol.

[CR115] Peirlinck M, Sack KL, De Backer P, Morais P, Segers P, Franz T, De Beule M (2019). Kinematic boundary conditions substantially impact in silico ventricular function. Int J Num Methods Biomed Eng.

[CR116] Pfaller MR, Hörmann JM, Weigl M, Nagler A, Chabiniok R, Bertoglio C, Wall WA (2019). The importance of the pericardium for cardiac biomechanics: from physiology to computational modeling. Biomech Model Mechanobiol.

[CR117] Phibbs B (2007). The human heart: a basic guide to heart disease.

[CR118] Pisano C, D’Amico F, Balistreri CR, Vacirca SR, Nardi P, Altieri C, Scioli MG, Bertoldo F, Santo L, Bellisario D, Talice M (2020). Biomechanical properties and histomorphometric features of aortic tissue in patients with or without bicuspid aortic valve. J Thoracic Dis.

[CR119] Po SS, Wang DW, Yang IC, Johnson JP, Nie L, Bennett PB (1999). Modulation of HERG potassium channels by extracellular magnesium and quinidine. J Cardiovas Pharmacol.

[CR120] Potse M, Dubé B, Richer J, Vinet A, Gulrajani RM (2006). A comparison of monodomain and bidomain reaction-diffusion models for action potential propagation in the human heart. IEEE Transac Biomed Eng.

[CR121] Prakosa A, Arevalo HJ, Deng D, Boyle PM, Nikolov PP, Ashikaga H, Blauer JJ, Ghafoori E, Park CJ, Blake RC, Han FT, MacLeod RS, Halperin HR, Callans DJ, Ranjan R, Chrispin J, Nazarian S, Trayanova NA (2018). Personalized virtual-heart technology for guiding the ablation of infarct-related ventricular tachycardia. Nat Biomed Eng.

[CR122] Propp A, Gizzi A, Levrero-Florencio F, Ruiz-Baier R (2020). An orthotropic electro-viscoelastic model for the heart with stress-assisted diffusion. Biomech Model Mechanobiol.

[CR123] Quarteroni A, Lassila T, Rossi S, Ruiz-Baier R (2017). Integrated Heart - Coupling multiscale and multiphysics models for the simulation of the cardiac function. Comput Methods Appl Mech Eng.

[CR124] Ramírez WA, Gizzi A, Sack KL, Guccione JM, Hurtado DE (2020). In-silico study of the cardiac arrhythmogenic potential of biomaterial injection therapy. Sci Rep.

[CR125] Rausch MK, Kuhl E (2013). On the effect of prestrain and residual stress in thin biological membranes. J Mech Phys Solids.

[CR126] Rausch MK, Famaey N, O’Brien Shultz T, Bothe W, Miller DC, Kuhl E (2013). Mechanics of the mitral valve: a critical review, an in vivo parameter identification, and the effect of prestrain. Biomech Model Mechanobiol.

[CR127] Rausch MK, Zollner AM, Genet M, Baillargeon B, Bothe W, Kuhl E (2017). A virtual sizing tool for mitral valve annuloplasty. Int J Num Methods Biomed Eng.

[CR128] Redfern WS, Carlsson L, Davis AS, Lynch WG, MacKenzie I, Palethorpe S, Siegl PKS, Strang I, Sullivan AT, Wallis R, Camm AJ, Hammond TG (2003). Relationships between preclinical cardiac electrophysiology, clinical QT interval prolongation and torsade de pointes for a broad range of drugs: Evidence for a provisional safety margin in drug development. Cardiovas Res.

[CR129] Rim Y, McPherson DD, Chandran KB, Kim H (2013). The effect of patient-specific annular motion on dynamic simulation of mitral valve function. J Biomech.

[CR130] Rodriguez EK, Hoger A, McCulloch AD (1994). Stress-dependent finite growth in soft elastic tissues. J Biomech.

[CR131] Rogers WJ, Shapiro EP, Weiss JL, Buchalter MB, Rademakers FE, Weisfeldt ML, Zerhouni EA (1991). Quantification of and correction for left ventricular systolic long-axis shortening by magnetic resonance tissue tagging and slice isolation. Circulation.

[CR132] Rose EA, Gelijns AC, Moskowitz AJ, Heitjan DF, Stevenson LW, Dembitsky W, Long JW, Ascheim DD, Tierney AR, Levitan RG, Watson JT, Meier P (2001). Long-term use of left ventricular assist device for end-stage heart failure. New England J Med.

[CR133] Rossi S, Ruiz-Baier R, Pavarino LF, Quarteroni A (2012). Orthotropic active strain models for the numerical simulation of cardiac biomechanics. Int J Num Methods Biomed Eng.

[CR134] Rotman OM, Bianchi M, Ghosh RP, Kovarovic B, Bluestein D (2018). Principles of TAVR valve design, modelling, and testing. Expert Rev Med Dev.

[CR135] Sack KL, Baillargeon B, Acevedo-Bolton G, Genet M, Rebelo N, Kuhl E, Klein L, Weiselthaler GM, Burkhoff D, Franz T, Guccione JM (2016). Partial LVAD restores ventricular outputs and normalizes LV but not RV stress distributions in the acutely failing heart in silico. Int J Artif Organs.

[CR136] Sack KL, Dabiri Y, Franz T, Solomon SD, Burkhoff D, Guccione JM (2018). Investigating the role of interventricular interdependence in development of right heart dysfunction during LVAD support: A patient-specific methods-based approach. Front Physiol.

[CR137] Sack KL, Aliotta E, Choy JS, Ennis DB, Davies N, Franz T, Kassab GS, Guccione JM (2020). Intra-myocardial alginate hydrogel injection acts as a left ventricular mid-wall constraint in swine. Acta Biomat.

[CR138] Saez P, Kuhl E (2016). Computational modeling of acute myocardial infarction. Comput Methods Biomech Biomed Eng.

[CR139] Sahli Costabal F, Hurtado DE, Kuhl E (2016). Generating Purkinje networks in the human heart. J Biomech.

[CR140] Sahli Costabal F, Concha FA, Hurtado DE, Kuhl E (2017). The importance of mechano-electrical feedback and inertia in cardiac electromechanics. Comput Methods Appl Mech Eng.

[CR141] Sahli Costabal F, Yao J, Kuhl E (2018). Predicting drug-induced arrhythmias by multiscale modeling. Int J Num Methods Biomed Eng.

[CR142] Sahli Costabal F, Zaman JAB, Kuhl E, Narayan SM (2018). Interpreting activation mapping of atrial fibrillation: a hybrid computational/physiological study. Ann Biomed Eng.

[CR143] Sahli Costabal F, Matsuno K, Yao J, Perdikaris P, Kuhl E (2019). Machine learning in drug development: characterizing the effect of 30 drugs on the qt interval using gaussian process regression, sensitivity analysis, and uncertainty quantification. Comput Methods Appl Mech Eng.

[CR144] Sahli Costabal F, Choy JS, Sack KL, Guccione JM, Kassab G, Kuhl E (2019). Multiscale characterization of heart failure. Acta Biomat.

[CR145] Sahli Costabal F, Perdikaris P, Kuhl E, Hurtado DE (2020). Multi-fidelity classification using Gaussian processes: accelerating the prediction of large-scale computational models. Comp Methods Appl Mech Eng.

[CR146] Sahli-Costabal F, Seo K, Ashley E, Kuhl E (2020). Classifying drugs by their arrhythmogenic risk using machine learning. Biophys J.

[CR147] Sahli Costabal F, Yang Y, Perdikaris P, Hurtado DE, Kuhl E (2020). Physics-informed neural networks for cardiac activation mapping. Front Phys.

[CR148] Sebastian R, Zimmerman V, Romero D, Sanchez-Quintana D, Frangi AF (2013). Characterization and modeling of the peripheral cardiac conduction system. IEEE Transac on Med Imaging.

[CR149] Segars WP, Bond J, Frush J, Hon S, Eckersley C, Williams CH, Feng J, Tward DJ, Ratnanather JT, Miller MI, Frush D, Samei E (2013). Population of anatomically variable 4D XCAT adult phantoms for imaging research and optimization. Med Phys.

[CR150] Segars WP, Mahesh M, Beck TJ, Frey EC, Tsui BM (2008). Realistic ct simulation using the 4D XCAT phantom. Med Phys.

[CR151] Segars WP, Norris H, Sturgeon GM, Zhang Y, Bond J, Minhas A, Tward DJ, Ratnanather JT, Miller MI, Frush D, Samei E (2015). The development of a population of 4D pediatric XCAT phantoms for imaging research and optimization. Med Phys.

[CR152] Segars WP, Veress AI, Sturgeon GM, Samei E (2019). Incorporation of the living heart model into the 4D XCAT phantom for cardiac imaging research. IEEE Transac Rad Plasma Med Sci.

[CR153] Sim K, Ershad F, Zhang Y, Yang P, Shim H, Rao Z, Lu Y, Thukral A, Elgalad A, Xi Y, Tian B (2020). An epicardial bioelectronic patch made from soft rubbery materials and capable of spatiotemporal mapping of electrophysiological activity. Nat Elect.

[CR154] Smith N, de Vecchi A, McCormick M, Nordsletten D, Camara O, Frangi AF, Delingette H, Sermesant M, Relan J, Ayache N, Krueger MW, Schulze WHW, Hose R, Valverde I, Beerbaum P, Staicu C, Siebes M, Spaan J, Hunter P, Weese J, Lehmann H, Chapelle D, Rezavi R (2011). euHeart: personalized and integrated cardiac care using patient-specific cardiovascular modelling. Interf Focus.

[CR155] Sodhani D, Reese S, Aksenov A, Soganci S, Jockenhovel S, Mela P, Stapleton SE (2018). Fluid-structure interaction simulation of artificial textile reinforced aortic heart valve: validation with an in-vitro test. J Biomech.

[CR156] Solomon SD, Shin SH, Shah A, Skali H, Desai A, Kober L, Maggioni AP, Rouleau JL, Kelly RY, Hester A, McMurray JJ, Pfeffer MA (2011). Aliskiren Study in Post-MI Patients to Reduce Remodeling (ASPIRE) Investigators Effect of the direct renin inhibitor aliskiren on left ventricular remodelling following myocardial infarction with systolic dysfunction. European Heart J.

[CR157] Sommer G, Schriefl AJ, Andra M, Sacherer M, Viertler C, Wolinski H, Holzapfel GA (2015). Biomechanical properties and microstructure of human ventricular myocardium. Acta Biomat.

[CR158] Stewart P, Aslanidi OV, Noble D, Noble PJ, Boyett MR, Zhang H (2009). Mathematical models of the electrical action potential of Purkinje fibre cells. Philos Transac Math Phys Eng Sci.

[CR159] Strauss DG, Selvester RH, Wagner GS (2011). Defining left bundle branch block in the era of cardiac resynchronization therapy. Am J Cardiol.

[CR160] Sturla F, Vismara R, Jaworek M, Votta E, Romitelli P, Pappalardo OA, Lucherini F, Antona C, Fiore GB, Redaelli A (2017). In vitro and in silico approaches to quantify the effects of the Mitraclip®system on mitral valve function. J Biomech.

[CR161] Takeuchi M, Nakai H, Kokumai M, Nishikage T, Otani S, Lang RM (2006). Age-related changes in left ventricular twist assessed by two-dimensional speckle-tracking imaging. J Am Soc Echocardiogr.

[CR162] Tawara S (1906). Das Reizleitungssystem des Säugetierherzens.

[CR163] Taylor CA, Fonte TA, Min JK (2013). Computational fluid dynamics applied to cardiac computed tomography for noninvasive quantification of fractional flow reserve: scientific basis. J Am CollCardiol.

[CR164] ten Tusscher KHWJ, Noble D, Noble PJ, Panfilov AV (2004). A model for human ventricular tissue. Am J Physiol Heart Circ Physiol.

[CR165] Timek TA, Nielsen SL, Lai DT, Tibayan F, Liang D, Daughters GT, Beineke P, Hastie T, Ingels NB, Miller DC (2004). Mitral annular size predicts alfieri stitch tension in mitral edge-to-edge repair. J Heart Valve Dis.

[CR166] Trayanova NA, Winslow R (2011). Whole-heart modeling: applications to cardiac electrophysiology and electromechanics. Circ Res.

[CR167] Trohman RG, Kim MH, Pinski SL (2004). Cardiac pacing: the state of the art. Lancet.

[CR168] Udelson JE, Stevenson LW (2016). The future of heart failure diagnosis, therapy, and management. Circulation.

[CR169] Vasconcellos EC, Clua EW, Fenton FH, Zamith M (2020). Accelerating simulations of cardiac electrical dynamics through a multi-GPU platform and an optimized data structure. Concurr Comput Pract Exp.

[CR170] Veress A, Segars WP, Samei E (2019) Utilizing deformable image registration to create new living human heart models for imaging simulation, SPIE Medical Imaging 2019: Phys Med Imaging, 10948

[CR171] Vergara C, Palamara S, Catanzariti D, Nobile F, Faggiano E, Pangrazzi C, Centonze M, Maines M, Quarteroni A, Vergara G (2014). Patient-specific generation of the purkinje network driven by clinical measurements of a normal propagation. Med Biol Eng Comput.

[CR172] Walker JC, Ratcliffe MB, Zhang P, Wallace AW, Fata B, Hsu EW, Saloner D, Guccione JM (2005). MRI-based finite-element analysis of left ventricular aneurysm. Am J Physiol Heart Circ Physiol.

[CR173] World Health Organization (2017) Cardiovascular diseases (CVDs) fact sheet. World Health Organization

[CR174] World Health Organization (2018) The top 10 causes of death. World Health Organization

[CR175] Wisneski AD, Wang Y, Deuse T, Hill AC, Pasta S, Sack KL, Yao J, Guccione JM (2020). Impact of aortic stenosis on myofiber stress: translational application of left ventricle-aortic coupling simulation. Front Physiol.

[CR176] Witzenburg CM, Holmes JW (2017). A comparison of phenomenologic growth laws for myocardial hypertrophy. J Elast.

[CR177] Young S, Bakic PR, Myers KJ, Jennings RJ, Park S (2013). A virtual trial framework for quantifying the detectability of masses in breast tomosynthesis projection data. Med Phys.

[CR178] Zhang Y, Wang VY, Morgan AE, Kim J, Handschumacher MD, Moskowitz CS, Levine RA, Ge L, Guccione JM, Weinsaft JW, Ratcliffe MB (2019). Mechanical effects of mitraclip on leaflet stress and myocardial strain in functional mitral regurgitation - a finite element modeling study. PLoS ONE.

[CR179] Zhou L, Bar-Cohen Y, Peck RA, Chirikian GV, Harwin B, Chmait RH, Pruetz JD, Silka MJ, Loeb GE (2017). Analytical modeling for computing lead stress in a novel epicardial micropacemaker. Cardiovas Eng Technol.

[CR180] Zygote Media Group Inc. Zygote Solid 3D Heart Generations I & II Development Report. Technical Development of 3D Anatomical Systems. 2014

